# Volatile Compounds from Grape Skin, Juice and Wine from Five Interspecific HybridGrape Cultivars Grown in Québec (Canada) for Wine Production

**DOI:** 10.3390/molecules200610980

**Published:** 2015-06-15

**Authors:** Amélie Slegers, Paul Angers, Étienne Ouellet, Tamara Truchon, Karine Pedneault

**Affiliations:** 1 Centre de Développement Bioalimentaire du Québec, La Pocatière, QC G0R 1Z0, Canada; E-Mails: amelie.slegers.1@ulaval.ca (A.S.); etienne.ouellet@cdbq.net (E.O.); 2 Department of Food Science, Université Laval, Quebec, QC G1V 0A6, Canada; E-Mails: paul.angers@fsaa.ulaval.ca (P.A.); tamara.truchon.1@ulaval.ca (T.T.)

**Keywords:** *Vitis*, cold climate viticulture, GC-MS-SPME, volatile compounds, Frontenac, Marquette, Marechal Foch, Sabrevois, St. Croix

## Abstract

Developed from crosses between *Vitis vinifera* and North American *Vitis* species, interspecific hybrid grape varieties are becoming economically significant in northern areas, where they are now extensively grown for wine production. However, the varietal differences between interspecific hybrids are not well defined, nor are the relationships between hybrid grape and wine composition, which causes significant drawbacks in the development of viticulture and winemaking of northern wines. In an effort to increase our understanding of interspecific hybrids, we have characterized the free volatile compounds profiles of berries (juice and skin) and wines of five red hybrid varieties (Frontenac, Marquette, Maréchal Foch, Sabrevois and St. Croix) grown in Québec (Canada), using GC-MS(TOF)-SPME. In grapes and wines, significantly higher levels of C_6_ and other fatty acid degradation products (FADP) were found in Frontenac, Maréchal Foch and Marquette. Terpenes were primarily located in the skin, with Marquette showing the highest level for these compounds. Both the level of terpenes and the level of FADP in grape were strongly correlated with their respective levels in wine, as demonstrated by the redundancy analyses. Nonanal, (*E*,*Z*)-2,6-nonadienal, β-damascenone, ethyl octanoate and isoamyl acetate showed the highest OAVs in the wines of the studied varieties.

## 1. Introduction

The chemistry of grape cultivars, especially varietal aroma, has a significant impact on the character of wine, its sensory perception and its quality. Varietal aroma can relate to a specific compound or to a small group of odoriferous molecules, but is usually attributable to the contribution of several volatile compounds occurring in grapes, in proportions that differ from one variety to another [1]. In *Vitis vinifera*, many varietal volatile compounds have been identified, including monoterpenes, responsible for the typical aroma of Muscat and other aromatic varieties, and rotundone, contributing to the pepper aroma often found in Syrah [1,2,3].

Interspecific hybrid grapes are crosses between *Vitis vinifera* and North American native grapevine species such as *V. riparia*, *V. labrusca*, and/or *V. rupestris*. Recently, breeding programs have been created in northern U.S. and Canadian universities to produce cold-hardy, disease-tolerant hybrids with high enological potential, in order to meet the needs for locally grown and sustainable wine production in northern areas. In the past 20 years, hybrid varieties such as Frontenac and Marquette have been released on the market and are now extensively planted in Eastern Canada, and Midwestern and North-Eastern United States [4,5,6]. Despite its relatively recent implementation, the hybrid wine industry has contributed over 400 million USD $ to the economy of Midwestern and North-Eastern United States in 2011, creating more than 12,000 jobs in the area, and continues to grow significantly year over year [7]. The Québec wine industry, the third largest in Canada, was initially oriented towards French hybrid varieties such as Maréchal Foch and Vidal, which are still highly planted, but cold-tolerant interspecific hybrids such as Frontenac, Sabrevois and St. Croix are also popular among local growers [6,8]. The cold-climate wine industry currently shows significant progress in Eastern Canada, with consumers increasing their interest for locally produced hybrid wines.

Despite its fast development, the hybrid wine industry faces significant challenges, many of them resulting from the unique chemistry of hybrid grapes. For instance, hybrid grape varieties are known to have high anthocyanin content and specific anthocyanin profiles that cause higher color intensities in hybrid wines, often characterized by bluish tones [9,10,11]. The low concentration, extractability, and degree of polymerization of their tannins also pose significant challenges to northern winemakers [12]. Additionally, because they are grown under northern conditions, interspecific hybrids generally have high titratable acidity [4].

One of the main issue with interspecific hybrid varieties is their particular bouquet, which led to the assumption—founded or not, that interspecific hybrids produce low-quality wines. Hence, the first published studies on the volatile compounds of interspecific hybrids mostly focused on the so-called “foxy” compounds such as methyl anthranilate, furaneol (2,5-dimethyl-4-hydroxy-2,3-dihydro-3-furanone), and *o*-aminoacetophenone [13,14]. Most of these compounds were later found to be mainly attributable to *Vitis labrusca* and showed fewer occurrence in other American *Vitis* species [15]. Recent studies on the volatile compounds of interspecific hybrids focused on the determination of impact odorants using GC-O/MS in table wine and icewine made from old French hybrid varieties such as Seyval Blanc [16] and Vidal [16,17], on the sensory properties of La Crescent wines [18], and on the relationship between sensory and chemical composition of Solaris wines [19]. In red interspecific hybrids, the analysis of impact odorant and sensory properties of commercial wines made from the *V. riparia*-based hybrid Frontenac allowed the identification of key volatiles compounds that may be variety-related, including linalool, hexanol, methyl salicylate, and eugenol [20], and provided insights on the sensory perception of Frontenac wines, which were found to exhibit cherry, black berry, green, floral and spicy attributes [21]. The instrumental characterization of Maréchal Foch wines made from grapes obtained after different viticulture treatments allowed the detection of several grape-specific compounds including terpenoids (citronellol, α-terpineol, β-damascenone) and C_6_ compounds (*i.e.*, (*Z*)-3-hexenol, hexanol) [22].

Despite these studies, the contribution of viticulture and winemaking to the aroma of interspecific hybrid wines, especially those produced under northern condition, is still poorly understood, and the lack of in-depth research on the biochemistry of these grapes prevent any further improvement with regards to the development of well-adapted viticulture and winemaking practices for northern wine production. Hence, a few studies focused on the relations between grape and wine volatile composition in interspecific hybrids, especially with regards to volatile compounds. In previous work, we reported the evolution of free volatile compounds during berry ripening of the *V. riparia*-based interspecific hybrids Frontenac and Marquette [4]. In 2011, Mansfield *et al.* traced some relationships between Frontenac wines and Frontenac juice but, according to the authors, the method used to extract volatile compounds from juice was not sufficiently efficient to quantify potentially significant grape-related compounds [20]. Yet such studies would provide valuable understanding on how to optimize interspecific hybrid grape quality from the field to the finished wine, especially because evidence is building that traditional winemaking practices developed for *V. vinifera* may not be appropriate for interspecific hybrid winemaking [23].

In an effort to evaluate the relationship between grape and wine volatile compounds, we have characterized the free volatile compounds profiles of berries and wines of five red interspecific hybrids (Frontenac, Marquette, Maréchal Foch, Sabrevois and St. Croix) extensively grown in Quebec, using GC-MS(TOF)-SPME. Grape samples were harvested according to local commercial practices [6], and juice and berry skin were analyzed separately to evaluate the potential contribution of skin maceration to the overall aroma of interspecific hybrid wines. The results allowed the differentiation of the interspecific hybrid cultivars based on their varietal character, and clear relationships could be traced between the level of certain volatile compounds in grapes, and the concentration of specific compounds in the wines.

## 2. Results

### 2.1. Juice and Wine Basic Metrics

Grapes were sampled at commercial harvest, according to the total soluble solid level usually targeted for the analyzed varieties [6]. Therefore, significant differences were found between varieties for both juices and wines metrics (Table 1). Frontenac and Marquette showed the highest TSS (23.8 and 23.7 °Brix respectively) compared to other varieties. Frontenac juice showed the highest TA (17.5 g/L tartaric acid eq.), whereas juices from St. Croix and Maréchal Foch showed the lowest (9.0 and 10.3 g/L tartaric acid eq. respectively). Sabrevois and St. Croix had the highest berry weight compared to Frontenac, Maréchal Foch and Marquette. In Maréchal Foch and St. Croix, skin accounted for a higher proportion of berry weight at 18.4% and 16.4% *w*/*w* FW, based on berry FW, respectively, compared to the other grape varieties under study.

In agreement with juice metrics, Frontenac wine showed the highest TA (8.58 g/L tartaric acid eq.) and a lower pH (3.42) compared to the other analyzed varieties. Wines showed differences in alcohol content, which correlated with the TSS content of the respective juices. Thus, the alcohol level in wines made from Frontenac, Maréchal Foch and Marquette grapes ranged from 12.3 (Maréchal Foch) to 13.4% *v*/*v* (Frontenac), whereas the alcohol level of the wines made from Sabrevois and St. Croix were 9.9% and 10.9% *v*/*v*, respectively.

### 2.2. Free Volatile Compounds from Grapes

Five classes of free volatile compounds were characterized in the juice and the skin of the interspecific hybrid varieties under study: (1) C_6_ and other fatty acids degradation products (FADP); (2) grape-derived fatty acid ethyl esters (FAEE); (3) terpenes; (4) C_13_-norisoprenoids; and (5) grape-derived volatile phenols and other benzene derivatives (Table 2 and Table 3). Among the eighteen FADP compounds identified in the juice, C_6_ aldehydes (hexanal, (*Z*)-3-hexenal, (*E*)-2-hexenal) and C_6_ alcohols (hexanol, (*Z*)-3-hexenol and (*E*)-2-hexenol) represented the largest proportion of total quantified volatiles in Frontenac, Maréchal Foch and Marquette. All varieties had higher FADP levels in their juice, with values ranging from 273 (St. Croix) to 3205 μg/L (Maréchal Foch) compared to 77 (St. Croix) to 525 μg/kg (Marquette) in their skin, with Sabrevois and St. Croix showing the lowest levels of C_6_ in both their juice and skin, compared to the other varieties. Significant levels of volatile phenols and other benzene derivatives (444 and 357 μg/L juice, respectively), principally 2-phenylacetaldehyde were found in Sabrevois and St. Croix juice. With the exception of Maréchal Foch and Sabrevois that contained significant amounts of FAEE in their juice (125 μg/L and 265 μg/L, respectively), other volatile compound classes, including terpenes and C_13_-norisoprenoids, showed much lower levels than other compound classes analyzed. Of interest, Marquette grapes showed the highest level of free terpenes in juice (4.73 μg/L) and in berry skin (10.5 μg/kg).

**Table 1:** Berry and juice quality attributes of the interspecific hybrid grape Frontenac, Maréchal Foch, Marquette, Sabrevois, and St. Croix harvested in the province of Québec (Canada), during the season 2012, and basic chemical composition of the produced wines.

Grape Variety	Berry			Juice				Wine				
	Fresh Weight (g/berry)	Skin Proportion (% *w*/*w* FW)	Juice Yield (% *w*/*w* FW)	Total Soluble Solids (°Brix)	Titratable Acidity (g/L Tartaric Acid eq.)	pH	Yeast Assimiliable Nitrogen (mg/L)	Alcohol Content (% *v*/*v*)	Titratable Acidity (g/L Tartaric Acid eq.)	pH	Glycerol (g/L)	Volatile Acidity (mg/L Acetic Acid eq.)
Frontenac	1.25 a ^1^	12.0 a	82.3 b	23.8 b	17.5 b	3.10 a	266 b	13.4 c	8.58 b	3.42 a	1.94 a	0.47 a
Maréchal Foch	1.23 a	18.4 b	75.7 a	21.6 ab	10.3 a	3.18 a	108 a	12.3 bc	5.90 a	3.74 ab	1.85 a	0.54 a
Marquette	1.17a	14.7 ab	78.6 ab	23.7 b	13.1 ab	3.09 a	210 ab	13.2 c	5.71 a	3.89 b	1.94 a	0.52 a
Sabrevois	1.81 b	14.0 ab	79.4 ab	18.6 a	13.4 ab	3.16 a	221 ab	9.90 a	6.11 a	3.69 ab	1.98 a	0.62 a
St. Croix	1.82 b	16.4 b	78.1 ab	19.4 a	9.0 a	3.21 a	129 a	10.9 ab	5.73 a	3.86 b	1.80 a	0.50 a

^1^ Values listed are the mean of six to eight replicates per grape variety. Values on the same column followed by a different letter are significantly different according to Tukey’s least significant difference test (*p* ≤ 0.05).

**Table 2:** Free volatile compound (μg/L) from the juice of the interspecific hybrid grape varieties Frontenac, Maréchal Foch, Marquette, Sabrevois, and St. Croix harvested in the province of Québec (Canada), during the season 2012.

Compound	Frontenac	Maréchal Foch	Marquette	Sabrevois	St. Croix
*Fatty Acid Degradation Products*					
hexanal	41.3 ± 43.5 ab ^1^	101 ± 99 ab	136 ± 132 b	8.34 ± 9.06 ab	3.26 ± 1.81 a
(*Z*)-3-hexenal	66.7 ± 40.2 bc	72.4 ± 50.8 c	48.5 ± 36.5 abc	9.55 ± 8.51 ab	1.37 ± 1.49 a
(*E*)-2-hexenal	808 ± 510 bc	927 ± 455 c	693 ± 536 abc	228 ± 234 ab	18.8 ± 16.8 a
2-octanone	0.09 ± 0.05 a	0.13 ± 0.11a	0.09 ± 0.10 a	0.02 ± 0.03 a	0.10 ± 0.12 a
1-octen-3-one	tr	0.01 ± 0.01	tr	tr	tr
(*E*)-2-heptenal	0.58 ± 0.32 ab	1.40 ± 0.92 b	0.73 ± 0.56 ab	0.66 ± 0.46 ab	0.11 ± 0.26 a
hexanol	262 ± 267 ab	784 ± 300 c	623 ± 138 bc	64.8 ± 20.5 a	111 ± 133 a
(*Z*)-3-hexenol	64.3 ± 25.5 c	25.0 ± 13.9 ab	55.1 ± 32.8 bc	23.7 ± 8.5 ab	18.5 ± 6.0 a
(*E*,*E*)*-*2,4-hexadienal	28.7 ± 19.4 ab	41.4 ± 30.0 b	25.0 ± 22.7 ab	2.23 ± 2.59 a	1.14 ± 0.44 a
(*E*)-2-hexenol	840 ± 384 b	1 111 ± 510 b	579 ± 219 ab	149 ± 47 a	81.5 ± 66.1 a
1-octen-3-ol	0.51 ± 0.17 a	1.56 ± 1.27 b	0.81 ± 0.32 ab	0.58 ± 0.19 ab	0.41 ± 0.26 a
heptanol	tr	0.24 ± 0.61	tr	tr	tr
(*E*,*Z*)*-*2,4-heptadienal	0.40 ± 0.12 b	0.41 ± 0.05 b	0.28 ± 0.17 ab	0.22 ± 0.18 ab	0.08 ± 0.13 a
(*E*,*E*)*-*2,4-heptadienal	2.13 ± 0.48 b	2.28 ± 0.38 b	1.54 ± 0.88 ab	1.62 ± 0.90 ab	0.84 ± 0.78 a
decanal	0.94 ± 0.01 a	0.95 ± 0.02 a	0.76 ± 0.42 a	0.62 ± 0.48 a	0.82 ± 0.36 a
(*E*,*Z*)*-*2,6-nonadienal	0.31 ± 0.19 ab	0.41 ± 0.24 b	0.15 ± 0.14 ab	0.27 ± 0.04 ab	0.13 ± 0.09 a
2-undecanone	0.27 ± 0.21 a	0.33 ± 0.28 a	0.59 ± 0.73 a	0.49 ± 0.46 a	1.09 ± 1.13 a
hexanoic acid	38.8 ± 17.9 a	135 ± 109 b	55.4 ± 33.3 ab	32.9 ± 25.3 a	34.3 ± 46.8 a
*Sum*	2 176 ± 679 b	3 205 ± 915 c	2 220 ± 799 bc	523 ± 300 a	273 ± 188 a
*Ethyl Esters*					
ethyl propanoate	0.36 ± 0.90 a	14.1 ± 11.6 b	0.03 ± 0.06 a	13.6 ± 7.76 b	0.05 ± 0.08 a
ethyl 2-methylpropanoate	0.21 ± 0.39 a	0.77 ± 2.04 a	nd	nd	nd
ethyl butanoate	0.34 ± 0.97 a	71.7 ± 49.0 a	tr	196 ± 97.8 b	tr
ethyl 2-methylbutanoate	nd	1.02 ± 0.76 a	nd	3.20 ± 1.58 b	nd
ethyl 3-methylbutanoate	nd	0.22 ± 0.22 a	nd	0.17 ± 0.18 a	nd
ethyl (*E*)-2-butenoate	0.05 ± 0.12 a	22.4 ± 23.1 b	tr	30.1 ± 13.9 b	0.02 ± 0.03 a
ethyl hexanoate	0.01 ± 0.02 a	14.5 ± 11.3 b	tr	19.5 ± 11.4 b	tr
ethyl octanoate	tr	tr	tr	2.09 ± 3.54 a	2.09 ± 5.50 a
*Sum*	0.97 ± 1.35 a	125 ± 90 b	0.03 ± 0.06 a	265 ± 126 c	2.16 ± 5.47 a
*Terpenes*					
β-myrcene	1.17 ± 0.96 a	1.60 ± 0.71 a	1.92 ± 0.06 a	1.24 ± 0.96 a	0.79 ± 0.98 a
(*R*)-(+)-limonene	0.12 ± 0.05 a	0.11 ± 0.06 a	0.12 ± 0.09 a	0.08 ± 0.04 a	0.10 ± 0.05 a
linalool	0.78 ± 0.11 a	0.86 ± 0.20 a	1.64 ± 0.87 b	0.62 ± 0.13 a	0.51 ± 0.23 a
α-terpineol	tr	tr	0.96 ± 1.20 b	0.10 ± 0.22 a	tr
β-citronellol	nd	tr	tr	0.01 ± 0.01	tr
nerol	tr	nd	0.09 ± 0.11 a	nd	0.10 ± 0.16 a
*Sum*	2.07 ± 0.97 a	2.57 ± 0.88 a	4.73 ± 2.03 b	2.05 ± 0.75 a	1.51 ± 0.90 a
*C_13_-Norisoprenoids*					
β-damascenone	3.21 ± 1.76 a	1.62 ± 0.85 a	6.00 ± 6.77 a	1.56 ± 1.26 a	2.68 ± 1.61 a
α-ionone	0.51 ± 0.39 a	0.56 ± 0.30 a	1.03 ± 0.96 a	0.66 ± 0.52 a	1.38 ± 0.99 a
α-ionol	1.86 ± 0.92 a	2.03 ± 0.57 a	3.54 ± 3.12 a	2.13 ± 0.92 a	4.86 ± 4.24 a
β-ionone	0.10 ± 0.04 a	0.20 ± 0.09 a	0.11 ± 0.03 a	0.06 ± 0.01 a	0.05 ± 0.02 a
*Sum*	5.68 ± 2.93 a	4.41 ± 1.59 a	10.7 ± 10.8 a	4.41 ± 2.66 a	8.97 ± 6.59 a
*Volatile Phenols And Benzene Derivatives*					
2-phenylacetaldehyde	2.24 ± 4.37 a	2.04 ± 4.50 a	3.75 ± 5.90 a	413 ± 183 b	298 ± 182 b
phenethyl acetate	0.02 ± 0.02 a	0.74 ± 1.54 a	0.01 ± 0.01 a	0.06 ± 0.08 a	0.23 ± 0.32 a
2-phenylethanol	4.72 ± 9.40 a	11.3 ± 23.3 a	0.90 ± 0.40 a	27.2 ± 29.9 a	57.2 ± 61.1 a
eugenol	0.49 ± 0.32 ab	0.30 ± 0.28 a	0.33 ± 0.31ab	0.75 ± 0.17 b	0.16 ± 0.27 a
*p-v*inylguaiacol	1.34 ± 1.41a	0.35 ± 0.54 a	0.22 ± 0.38 a	2.91 ± 2.78 a	1.34 ± 3.20 a
*Sum*	8.81 ± 12.7 a	14.7 ± 24.0 a	5.21 ± 6.02 a	444 ± 194 b	357 ± 141 b
*Others*					
isoamyl acetate	0.10 ± 0.15 a	1.15 ± 1.76 a	0.02 ± 0.04 a	0.53 ± 0.48 a	0.89 ± 1.90 a
isoamyl alcohol	tr	tr	tr	tr	0.44 ± 1.03

^1^ Values are listed as mean ± standard deviation of six to eight samples per grape variety. Values on the same line followed by a different letter are significantly different according to Tukey’s least significant difference test (*p* ≤ 0.05). When a compound could be quantified in more than 50% of samples, but found to be under LOQ in the remaining samples, the mean was calculated using all samples, with the quantification value for samples above LOQ, and with LOQ/2 in samples containing levels below LOQ; nd: Not detected; tr: Compound was found below LOQ in most samples.

**Table 3:** Free volatile compounds (μg/kg berry) from berry skin of the interspecific hybrids Frontenac, Maréchal Foch, Marquette, Sabrevois, and St. Croix harvested in the province of Québec (Canada), during the season 2012.

Compound	Frontenac	Maréchal Foch	Marquette	Sabrevois	St. Croix
*Fatty Acid Degradation Products*					
hexanal	27.7 ± 9.58 a	126 ± 51.0 ab	222 ± 93 b	53.5 ± 39.7 ab	36.3 ± 22.5 a
(*E*)-2-hexenal	52.5 ± 28.7 ab	174 ± 138 ab	270 ± 143 b	55.1 ± 25.5 ab	29.4 ± 12.3 a
hexanol	9.29 ± 3.01 a	17.3 ± 5.14 a	15.9 ± 2.39 a	13.2 ± 4.10 a	9.47 ± 3.31 a
(*Z*)-3-hexenol	9.35 ± 1.67 ab	8.96 ± 0.61 a	12.3 ± 2.30 b	9.08 ± 0.82 ab	9.20 ± 1.57 a
(*E*)-2-hexenol	2.73 ± 2.22 a	5.19 ± 2.30 ab	4.06 ± 3.21 b	2.25 ± 2.00 a	0.61 ± 0.45 a
1-octen-3-ol	1.38 ± 0.05 a	1.39 ± 0.17 a	1.21 ± 0.11 a	1.28 ± 0.22 a	1.19 ± 0.10 a
hexanoic acid	tr	tr	tr	tr	tr
*Sum*	102 ± 34 a ^1^	333 ± 167 b	525 ± 235 b	115 ± 63 a	77.3 ± 38.4 a
*Ethyl Esters* ^2^					
ethyl 2-methylbutanoate	nd	tr	nd	0.12 ± 0.003	nd
ethyl butanoate	0.58 ± 0.24 a	8.26 ± 9.26 b	tr	13.8 ± 5.47 b	0.55 ± 0.48 a
ethyl (*E*)-2-butenoate	tr	3.35 ± 2.42 a	nd	1.79 ± 1.37 b	nd
ethyl hexanoate	tr	tr	tr	tr	tr
*Sum*	0.49 ± 0.44 a	10.7 ± 10.9 bc	0.20 ± 0.23 ab	15.8 ± 5.87 c	0.47 ± 0.48 a
*Terpenes*					
β-myrcene	0.21 ± 0.11 a	0.31 ± 0.06 a	1.64 ± 0.67 b	0.49 ± 0.28 a	0.21 ± 0.04 a
*R*-(+)-limonene	0.53 ± 0.05 a	0.53 ± 0.03 a	0.96 ± 0.43 b	0.63 ± 0.20 ab	0.51 ± 0.03 a
linalool	0.47 ± 0.12 a	0.61 ± 0.26 a	6.94 ± 5.91 b	3.48 ± 1.36 ab	0.91 ± 0.55 a
α-terpineol	tr	tr	tr	tr	tr
β-citronellol	0.06 ± 0.05 a	*0.*17 ± 0.10 ab	0.25 ± 0.08 c	0.37 ± 0.12 bc	nd
nerol	0.64 ± 0.09 a	0.67 ± 0.04 a	0.72 ± 0.06 a	0.70 ± 0.07 a	0.66 ± 0.07 a
*Sum*	1.88 ± 0.25 a	2.21 ± 0.40 a	10.5 ± 7.04 b	5.67 ± 1.86 a	2.33 ± 0.66 a
*C_13_-Norisoprenoids*					
β-damascenone	25.0 ± 19.7 a	16.0 ± 5.67 a	11.6 ± 1.88 a	18.9 ± 6.43 a	34.3 ± 11.7 a
β-ionone	0.23 ± 0.04 a	0.28 ± 0.08 a	0.28 ± 0.08 a	0.29 ± 0.15 a	0.31 ± 0.10 a
*Sum*	25.2 ± 19.7 ab	16.3 ± 5.72 ab	11.8 ± 1.90 a	19.2 ± 6.54 ab	34.6 ± 11.8 b
*Volatile Phenols And Benzene Derivatives*					
2-phenylacetaldehyde	tr	tr	tr	18.7 ± 12.7 a	15.7 ± 6.74 a
2-phenylethanol	18.3 ± 25.3 a	16.7 ± 18.9 a	17.5 ± 11.9 a	332 ± 95.6 b	29.7 ± 18.4 a
eugenol	tr	nd	nd	0.73 ± 0.36	nd
*Sum*	18.0 ± 25.8 a	19.0 ± 16.9 a	17.6 ± 12.0 a	351 ± 100 b	45.4 ± 23.2 a

^1^ Values are listed as mean ± standard deviation of six to eight samples per grape variety. Values on the same line followed by a different letter are significantly different according to Tukey’s least significant difference test (*p* ≤ 0.05). When a compound could be quantified in more than 50% of samples, but found to be under LOQ in the remaining samples, the mean was calculated using all samples, with the quantification value for samples above LOQ, and with LOQ/2 in samples containing levels below LOQ; ^2^ Other fatty acid ethyl esters such as ethyl propanoate, ethyl 2-methylpropanoate, ethyl 3-methylbutanoate, and ethyl octanoate were not detected in berry skin extract; nd: Not detected; tr: Compound was found below LOQ in most samples.

### 2.3. Wine Volatile Compounds

Volatile compounds analysis of wines produced from interspecific hybrid grapes resulted in the quantification of 39 compounds, including grape-derived compounds such as FADP, C_13_-norisoprenoids, and terpenes, as well as fermentation-derived compounds such as aliphatic and aromatic esters, alcohols, and free fatty acids (FFA) (Table 4). Additional compounds including, methyl anthranilate, methyl salicylate, *o*-aminoacetophenone, and isoeugenol were detected and tentatively quantified, but their occurrence in the wine samples was inconsistent and/or below the limit of quantification, which is in agreement with known limitation of SPME.

The occurrence of fatty acid degradation products, which mostly included hexanol, (*Z*)*-*3-hexenol and nonanal, was significantly higher in Marquette (2155 μg/L) and Maréchal Foch (2622 μg/L), compared to Frontenac (1244 μg/L), Sabrevois (976 μg/L) and St. Croix (422 μg/L). Marquette wines also showed significantly higher concentrations of terpenes (β-citronellol, linalool, geraniol, 98.6 μg/L) compared to the other wines. In contrast, the concentration of β-damascenone was similar in all wines. The concentration of eugenol was significantly higher in Sabrevois wines (23 μg/L) compared to the other varieties.

**Table 4:** Concentration, odor perception threshold and odor activity value (OAV) of free volatile compounds from wine made from the interspecific hybrid varieties Frontenac, Maréchal Foch, Marquette, Sabrevois and St. Croix harvested in the province of Québec (Canada), during the season 2012. Concentrations and odor perception thresholds are in μg/L, unless otherwise indicated.

Compound	Odor Perception Threshold (ref.) ^1^	Frontenac		Maréchal Foch		Marquette		Sabrevois		St.Croix	
		means ± sd	OAV	means ± sd	OAV	means ± sd	OAV	means ± sd	OAV	means ± sd	OAV
*Fatty Acid Degradation Products*											
hexanal	5 (1) *	4.80 ^2^ ± 1.16 ab	1	6.27 ± 1.07 b	1	6.22 ± 0.74 b	1	5.42 ± 2.15 ab	1	3.82 ± 0.93 a	1
hexanol	8000 (2)	1098 ± 304 b	<0.5	2467 ± 770 c	<0.5	1795 ± 140 c	<0.5	853 ± 206 ab	<0.5	331 ± 72 a	<0.5
(*E*)-3-hexenol	1000 (3) #	17.2 ± 5.4 a	<0.5	46.6 ± 15.8 b	<0.5	18.2 ± 1.8 a	<0.5	11.4 ± 3.3 a	<0.5	5.0 ± 0.8 a	<0.5
(*Z*)-3-hexenol	400 (2)	82.3 ± 41.9 a	<0.5	66.0 ± 28.0 a	<0.5	282 ± 109 b	1	71.7 ± 30.9 a	<0.5	50.5 ± 22.5 a	<0.5
nonanal	1 (4) *	40.1 ± 16.8 a	40	34.8 ± 14.5 a	35	52.2 ± 25.4 a	52	33.4 ± 7.0 a	33	30.0 ± 7.6 a	30
(*E*,*Z*)-2,6-nonadienal	0.01 (5) *	1.26 ± 0.28 a	126	1.44 ± 0.18 a	144	1.15 ± 0.30 a	115	1.13 ± 0.34 a	113	1.19 ± 0.26 a	119
*Sum*		1244 ± 326 b		2622 ± 797 c		2155 ± 239 c		976 ± 232 ab		422 ± 74 a	
*C*_13_*-Norisoprenoids*											
β-damascenone	0.05 (2)	3.99 ± 1.77 b	80	2.25 ± 0.71 ab	45	2.47 ± 0.85 ab	49	1.72 ± 0.63 a	34	3.38 ± 1.84 ab	68
*Terpenes*											
β-myrcene	14 (6) *	0.63 ± 0.42 a	<0.5	1.11 ± 0.59 a	<0.5	2.74 ± 0.95 b	<0.5	0.98 ± 0.49 a	<0.5	0.83 ± 0.38 a	<0.5
*p*-cymenene	-	0.56 ± 0.55 a	-	0.56 ± 0.50 a		1.72 ± 1.12 b		0.95 ± 0.55 ab		0.46 ± 0.39 a	
linalool	25.2 (7)	7.49 ± 1.26 a	<0.5	9.17 ± 3.49 a	<0.5	36.2 ± 7.7 b	1	8.84 ± 2.41 a	<0.5	7.07 ± 2.58 a	<0.5
α-terpineol	250 (2)	0.66 ± 0.24 a	<0.5	0.87 ± 0.21 ab	<0.5	2.37 ± 0.59 c	<0.5	1.40 ± 0.41 b	<0.5	0.70 ± 0.37 a	<0.5
β*-*citronellol	100 (2)	8.53 ± 3.50 a	<0.5	15.4 ± 4.0 a	<0.5	28.8 ± 6.2 b	<0.5	14.9 ± 6.5 a	<0.5	16.4 ± 6.0 a	<0.5
nerol	400 (8)	3.66 ± 1.12 a	<0.5	4.75 ± 2.84 a	<0.5	7.73 ± 2.06 a	<0.5	4.78 ± 3.26 a	<0.5	4.54 ± 2.48 a	<0.5
geraniol	30 (7)	2.03 ± 2.30 a	<0.5	6.61 ± 3.92 a	<0.5	19.1 ± 3.2 b	1	4.07 ± 4.40 a	<0.5	4.19 ± 3.59 a	<0.5
*Sum*		23.6 ± 6.6 a		38.5 ± 13.1 a		98.6 ± 13.3 b		35.9 ± 16.5 a		34.2 ± 12.7 a	
*Volatile Phenols*											
eugenol	6 (7)	4.34 ± 1.92 a	1	8.36 ± 2.31 a	1	6.80 ± 0.67 a	1	23.1 ± 4.79 b	4	6.00 ± 1.66 a	1
*p*-vinylguaiacol	40 (2)	8.39 ± 2.17 ab	<0.5	11.6 ± 3.1 b	<0.5	9.54 ± 1.80 b	<0.5	9.24 ± 2.71 b	<0.5	5.51 ± 0.83 a	<0.5
*Sum*		12.7 ± 3.7 a		20.0 ± 3.5 b		16.3 ± 2.1 ab		32.3 ± 6.3 c		11.5 ± 1.7 a	
*Ethyl Esters*											
ethyl 2-methylpropanoate	15 (7)	281 ± 81 a	19	281 ± 52 a	19	254 ± 40 a	17	283 ± 58 a	19	265 ± 63 a	18
ethyl butanoate	20 (2)	130 ± 91 ab	7	68.3 ± 48.6 a	3	327 ± 300 b	16	56.3 ± 44.5 a	3	49.8 ± 37.3 a	2
ethyl-2-methylbutanoate	18 (7)	5.00 ± 5.3 ab	<0.5	8.89 ± 5.7 ab	<0.5	3.16 ± 2.2 a	<0.5	12.6 ± 6.9 b	1	3.52 ± 3.4 a	<0.5
ethyl 3-methylbutanoate	3 (7)	12.1 ± 5.7 a	4	11.8 ± 3.0 a	4	11.0 ± 3.1 a	4	9.7 ± 0.9 a	3	9.0 ± 1.1 a	3
ethyl hexanoate	14 (7)	450 ± 241 ab	32	227 ± 146 a	16	803 ± 319 b	57	190 ± 146 a	14	179 ± 173 a	13
ethyl octanoate	5 (7)	1 128 ± 682 ab	226	781 ± 601 a	156	2 383 ± 519 b	477	643 ± 336 a	129	768 ± 421 ab	154
ethyl decanoate	200 (7)	5.56 ± 1.50 a	<0.5	19 ± 27 a	<0.5	445 ± 323 b	2	54 ± 29 a	<0.5	25 ± 50 a	<0.5
ethyl-3-hydroxyhexanoate	45 (9)	6.50 ± 3.01 a	<0.5	13.5 ± 5.3 b	<0.5	5.24 ± 1.04 a	<0.5	19.1 ± 7.7 b	<0.5	3.57 ± 1.15 a	<0.5
*Sum*		2114 ± 877 a		1397 ± 718 a		4226 ± 260 b		1217 ± 387 a		1432 ± 535 a	
*Phenolic Esters*											
ethyl phenylacetate	75 (10)	tr		36.5 ± 57.5 b		tr		9.39 ± 15.1 a		tr	
phenethyl acetate	250 (7)	19.4 ± 17.0 a	<0.5	30.3 ± 17.5 a	<0.5	10.4 ± 4.3 a	<0.5	23.1 ± 9.2 a	<0.5	36.5 ± 23.3 a	<0.5
ethyl dihydrocinnamate	1.6 (7)	2.41 ± 1.02 a	2	1.46 ± 0.67 a	1	4.80 ± 1.30 b	3	1.25 ± 0.42 a	1	2.37 ± 0.91 a	1
ethyl cinnamate	1.1 (7)	4.23 ± 1.91 a	4	3.58 ± 2.32 a	3	3.47 ± 2.02 a	3	3.88 ± 2.16 a	4	3.59 ± 2.22 a	3
ethyl vanillate	990 (11)	16.1 ± 7.8 a	<0.5	32.2 ± 14.6 b	<0.5	23.8 ± 5.0 ab	<0.5	9.57 ± 4.77 a	<0.5	11.4 ± 1.5 a	<0.5
*Sum*		42.2 ± 19.7 a		104 ± 70 b		42.4 ± 4.5 ab		47.2 ± 16.7 ab		53.9 ± 23.3 ab	
*Fatty Acids*											
hexanoic acid	420 (7)	1302 ± 1052 ab	3	850 ± 601 a	2	2142 ± 779 b	5	779 ± 306 a	2	506 ± 270 a	1
octanoic acid	500 (7)	892 ± 771 ab	2	214 ± 337 a	<0.5	1452 ± 548 b	3	273 ± 580 a	1	801 ± 855 ab	2
*Sum*		2194 ± 1792 ab		1064 ± 750 a		3594 ± 1324 b		1052 ± 828 a		1307 ± 1053 a	
*Other Fermentation Products*											
isobutyl acetate	6140 (9)	53.5 ± 90.6 a	<0.5	25.7 ± 52.2 a	<0.5	28.7 ± 44.5 a	<0.5	88.1 ± 154.8 a	<0.5	25.6 ± 41.7 a	<0.5
isoamyl acetate	30 (2)	1957 ± 1 404 a	65	1324 ± 405 a	44	1095 ± 938 a	37	1243 ± 299 a	41	1682 ± 820 a	56
hexyl acetate	26 (12)	2.82 ± 1.89 a	<0.5	3.28 ± 0.78 a	<0.5	2.32 ± 0.84 a	<0.5	1.89 ± 0.50 a	<0.5	1.77 ± 0.66 a	<0.5
ethyl lactate (mg/L)	100 (9)	85.3 ± 42.3 b	1	65.8 ± 13.4 ab	1	57.6 ± 20.9 ab	1	61.0 ± 17.4 ab	1	45.5 ± 15.3 a	<0.5
acetoin (mg/L)	150 (12)	21.3 ± 14.7 b	<0.5	7.91 ± 6.7 ab	<0.5	11.2 ± 9.3 ab	<0.5	2.61 ± 1.9 a	<0.5	2.96 ± 6.1 a	<0.5
butyrolactone	100,000 (9)	320 ± 34 a	<0.5	376 ± 42 a	<0.5	358 ± 8 a	<0.5	345 ± 42 a	<0.5	333 ± 58 a	<0.5
isobutanol	40,000 (2)	864 ± 261 a	<0.5	931 ± 207 a	<0.5	756 ± 106 a	<0.5	1 092 ± 266 a	<0.5	843 ± 351 a	<0.5
2-phenylethanol (mg/L)	14 (7)	47.4 ± 18 a	3	62.6 ± 11 a	4	48.0 ± 7.3 a	3	51.0 ± 9.2 a	4	55.6 ± 20 a	4

^1^ Odor perception thresholds in wine-like matrices, except for those followed by a star (*), which were measured in water, and those followed by hash (#), that were measured in dipropylene glycol; Thresholds were obtained from the following references: (1) Buttery *et al*., 1989 [24]; (2) Guth, 1997 [25]; (3) Hatanaka *et al*., 1992 [26]; (4) Guadagni *et al*., 1963 [27]; (5) Teranishi *et al*., 1974 [28]; (6) Buttery *et al*., 1968 [29]; (7) Ferreira *et al*., 2000 [30]; (8) Ribéreau-Gayon *et al*., 1975 [31]; (9) Zea *et al*., 2007 [32]; (10) Tat *et al*., 2007 [33]; (11) Culleré *et al*., 2004 [34]; (12) Etievant, 1991 [35]. ^2^ Values are listed as mean ± standard deviation of six to eight samples per grape variety. Values on the same line followed by a different letter are significantly different according to Tukey’s least significant difference test (*p* ≤ 0.05). When a compound could be quantified in more than 50% of samples, but found to be under LOQ in the remaining samples, the mean was calculated using all samples, with the quantification value for samples above LOQ, and with LOQ/2 in samples containing levels below LOQ; tr: Compound was found below LOQ in most samples.

Among fermentation related compounds, FAEE and FFA showed higher levels in Marquette wines (4200 and 3600 μg/L, respectively) compared to the wines of other varieties, but phenolic esters, including ethyl phenylacetate and ethyl vanillate, where more concentrated in the wines made from Maréchal Foch (104 μg/L).

### 2.4. Redundancy Analyses

**Figure 1:**
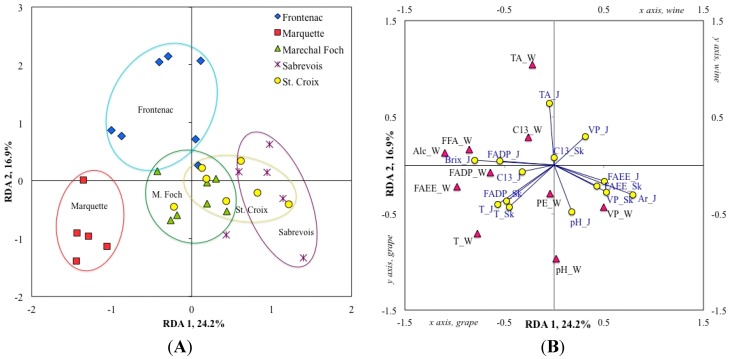
Redundancy analysis relating the chemical composition of the berry skin and juice (independent variables) to the chemical composition of the wines (dependant variables) made from the interspecific hybrid grape varieties Frontenac (blue diamonds), Marquette (red squares), Maréchal Foch (green triangles), Sabrevois (purple stars) and St. Croix (yellow circles). (**A**) Varieties samples plot (*n* = 32); (**B**) Biplot of grape (yellow circles, 14 variables) and wine (red triangles, 10 variables). The berry skin and juice variables are identified as follow: total soluble solids (Brix_J), titratable acidity (TA_J), pH (pH_J), sum of fatty acids degradation products in juice (FADP_J) and in skin (FADP_Sk), sum of terpenes in juice (T_J) and in skin (T_Sk), sum of C_13_-norisoprenoids in juice (C13_J) and in skin (C13_Sk), fatty acid ethyl esters in juice (FAEE_J) and in skin (FAEE_Sk), volatile phenols in juice (Ar_J, including 2-phenylacetaldehyde and 2-phenylethanol; VP_J, including eugenol and *p*-vinylguaiacol) and in skin (VP_Sk, including 2-phenylacetaldehyde, 2-phenylethanol, and eugenol). The wine variables are identified as follow: alcohol percentage (Alc_W), titratable acidity (TA_W), pH (pH_W), sum of fatty acids degradation products (FADP_W), sum of terpenes (T_W), volatile phenols (VP_W, including eugenol and *p*-vinylguaiacol), fatty acid ethyl esters (FAEE_W), free fatty acids (FFA_W, including hexanoic acid and octanoic acid), phenolic esters (PE_W), C_13_-norisoprenoids (C13_W, including only β-damascenone). For variables grouped as a sum of compounds that are not listed here (e.g., FADP, terpenes, C_13_-norisoprenoids, FAEE, Phenolic esters), see Table 1, Table 2 and Table 3 for the complete list of compounds grouped in each class.

A global redundancy analysis (RDA), using volatile compound grouped by classes (e.g., FADP, terpenes), was performed in order to evaluate the varietal characteristics of the interspecific hybrid varieties studied (Figure 1A), and to relate volatile compounds from grape juice and berry skin to the volatile compounds of the resulting wines (Figure 1B). This RDA model, significant to *p* ≤ 0.001 (Table 5), allowed the discrimination of Marquette from the other analyzed varieties and showed similarities between Sabrevois and St. Croix. The plot of RDA variables resulted in significant correlations between juice and wine technological parameters such as total soluble solids and alcohol percentage, as well as respective titratable acidity and pH of juice and wine (Figure 1B). Among volatile compounds, the sum of terpenes in both juice and skin were strongly correlated with the sum of terpenes in wine, and the level of FADP in juice was correlated with that of FADP in the wine. Both terpenes and FADP were characteristics of Marquette according to the sample plot (Figure 1A). The occurrence of volatile phenols in berry skin (VP_Sk; including 2-phenylacetaldehyde, 2-phenylethanol, and eugenol) and in the juice (Ar_J, including 2-phenylacetaldehyde and 2-phenylethanol) was correlated with the occurrence of volatile phenols and other benzene derivatives (VP_W, including eugenol and *p*-vinylguaiacol) in wine, which was characteristic of Sabrevois and St. Croix.

**Table 5:** Significance of the RDA models and canonical axes assessed by permutation tests (up to 1000 permutations allowed), and proportion of variance explained by each canonical axis (%), for the following RDAs: (1) Grouped compounds; (2) FADP; (3) Terpenes and C_13_-norisoprenoids; (4) Non-aromatic esters, acids and alcohols; (5) Aromatics.

Analysis	Anova on RDA (*p*-Value)	Anova on Canonical Axes		
		Canonical Axe	*p*-Value	Proportion of Variance Explained (%)
Grouped compounds ^1^	0.001	RDA1	0.001	24.2
		RDA2	0.001	16.9
FADP	0.13	RDA1	0.001	39.1
		RDA2	0.001	17.5
Terpenes and C_13_-norisoprenoids	0.001	RDA1	0.001	56.3
		RDA2	0.001	10.0
Non-aromatic esters, alcohols and acetates	0.57	RDA1	0.003	13.1
		RDA2	0.023	9.06
Aromatics	0.01	RDA1	0.001	13.5
		RDA2	0.011	8.79

^1^ The biplot of each RDA model are shown in the following figures: Figure 1B (Grouped compounds); Figure 2A (FADP); Figure 2B (Terpenes and C_13_-norisoprenoids); Figure 2C (Non-aromatic esters, alcohols and acetates); Figure 2D (Aromatics).

## 3. Discussion

Grape volatile compounds have been intensively studied in *V. vinifera* varieties in order to better understand the contribution of variety-specific volatile compounds to wine aroma [1,2,36,37,38,39]. The usual methodologies for such studies have been to quantify both free and bound volatile compounds from grapes, using enzymatic [40,41,42] or acid [43] hydrolysis to release bound precursors; or to analyze volatile compounds from monovarietal wines [20,44]. In the present study on the relationships between grape and wine volatile compounds in interspecific hybrid varieties, we analyzed free volatile compounds from both juice and berry skin, and from monovarietal wines made from our grape samples, and evaluated relationships between both using redundancy analyses.

### 3.1. Grapes

The grape varieties analyzed in this study showed significant differences in the total concentration of volatile compounds. Marquette and Maréchal Foch showed the highest concentration of combined juice and skin volatiles, at 2900–3200 μg/kg FW berries, respectively, whereas St. Croix showed the lowest concentration at 612 μg/kg FW berries. FADP, mainly C_6_ compounds, are significant contributors to both grape and wine volatile compounds [45,46,47,48]. In berries, 62%–95% of total FADP were located in the juice rather than berry skin. Overall, FADP accounted for over 93% of total quantified volatile compounds in Frontenac, Marquette and Maréchal Foch. High proportions of C_6_ had also been reported in hybrids of *V. thunbergii* X *V. vinifera* (98.1% of total volatile compounds), in hybrids of *V. amurensis* X *V. vinifera* (93.7% of total volatile compounds), and in *V. amurensis* species (98.4% of total volatile compounds) [49]. In contrast, Sabrevois and St. Croix showed lower proportions of FADP at 41%–42% of total quantified volatile compounds, in agreement with published data on hybrids of *V. labrusca* X *V. vinifera*, for which FADP accounted for 43% of C_6_, and for less than 25% in *V. labrusca* varieties [49]. Indeed, Sabrevois and St. Croix have the same parentage that includes 25% of *V. labrusca*, which may explain the low occurrence of C_6_ in these varieties [6]. C_6_ compounds are known to contribute to the aroma of many fruits and vegetables [50] and are major contributors to the varietal aroma of neutral grape varieties [51,52]. As demonstrated in the present study, as well as in previously published studies, the levels of FADP in grape juice varies from one grape variety to another [4,53,54], suggesting that grape genetics modulate lipid oxidation processes.

The levels of (*Z*)-3-hexenol and hexanal in the juice of the interspecific hybrids analyzed were similar to those from the juice of neutral *V. vinifera* varieties such as Pinot Noir (26–40 μg/L and 45–87 μg/L, respectively; measured by GC-MS-SBSE, [55]) and the red Spanish variety Serradelo (19–45 μg/L, for (*Z*)-3-hexenol, [53]). In contrast, the level of hexanol in our juice samples were generally higher than Pinot Noir (27–90 μg/L) but compared to the hexanol content of Serradelo (338–538 μg/L, [53]), although Maréchal Foch and Marquette showed higher concentrations for this compound (784 and 623 μg/L, respectively). Levels of two oxidation products of α-linolenic acid, (*E*)-2-hexenal and (*E*)-2-hexenol, were significantly higher in the juice of Frontenac, Maréchal Foch and Marquette (693–927 μg/L and 579–1100 μg/L, respectively) compared to the *V. vinifera* variety Pinot Noir grown in Oregon (United States) (35–43 and 288–590 μg/L, respectively, [55]). High concentration of unsaturated fatty acids are ubiquitous in plants grown under cold-climate, therefore it could be hypothesized that the high levels of certain C_6_ observed in cold-climate hybrids may relate to higher levels of unsaturated fatty acids. This hypothesis is supported by the fact that, in contrast with previous studies [54,56], we detected significant amounts of (*Z*)-3-hexenal (1.37–72.4 μg/L) in juice samples. (*Z*)-3-Hexenal is the immediate precursors of (*E*)-2-hexenal and (*E*)-2-hexenol through an enzymatic isomerization process, and is usually not reported in *V. vinifera* varieties [54,56]. Thus, the significant levels of (*Z*)-3-hexenal observed in interspecific hybrids may either relate to higher level of unsaturated fatty acids, to higher rate of oxidations and/or to lower rate of isomerization to (*E*)-2-hexenal and (*E*)-2-hexenol.

Terpenes are generally characteristic of the so-called “aromatic” grape varieties such as Muscat, Riesling, and Gewürztraminer [57], but they are also found in low amounts in neutral *Vitis vinifera* varieties at levels ranging between 16 (Cabernet Gernischt) and 36 μg/L (Cabernet Franc and Cabernet Sauvignon) in juice [45]. Their occurrence in *V. labrusca* and its hybrids, as well as in *V. riparia* grape varieties has been reported to be even lower than the values reported for non-aromatic *V. vinifera* varieties [49,58]. The results from the present study generally agree with the aforementioned studies, since the terpene concentration ranged from 2 to 11 μg/kg berry in the skin and accounted for less than 0.5% of volatiles in juice for most varieties analyzed, except for Marquette, where a higher proportion (1.1%) was found, hence agreeing with previous results we published on this variety [4]. Despite its strong cold-hardiness [59], Marquette includes a significant proportion *V. vinifera* in its parentage (63.1%) [6] that could partially explain the higher level of terpenes found in this variety. Similar to terpenes, C_13_-norisoprenoids were primarily concentrated in the skin (79%–94% of grape C_13_-norisoprenoids) rather than in the juice (6%–21% of grape C_13_-norisoprenoids). β-Damascenone was the main C_13_ found both in berry juice and skin, and in wine. No significant differences were found between varieties for the concentration of C_13_-norisoprenoids in their juice and skin, likely because large variations were observed among samples from a same variety.

2-Phenylacetaldehyde and 2-phenylethanol represented a significant part of grape volatile compounds in the juice from Sabrevois and St. Croix, at 37.7% and 51.2% of total quantified grape aroma, respectively. The juice of both these varieties showed higher levels of 2-phenylacetaldehyde compared to *V. vinifera* and other *Vitis* varieties, where trace levels up to 3.7 μg/kg berries have been found, although *V. labrusca* hybrids generally showed higher levels than hybrids bred from other *Vitis* species for this compound [45,49]. However, the values reported in the aforementioned studies were obtained in grape homogenate or in juice extracted from grape homogenate as opposed to the present study where juice was extracted with little skin contact, therefore suggesting that the high level of 2-phenylacetaldehyde found in the juice of Sabrevois and St. Croix may relate to the extraction process we used for juice extraction, which minimized skin damage. 2-Phenylacetaldehyde is the immediate precursor in the biosynthesis of 2-phenylethanol from the amino acid phenylalanine [50]. The present results on Sabrevois show high levels of 2-phenylacetaldehyde in the juice and of 2-phenylethanol in the skin. This may suggest that biosynthesis of 2-phenylethanol from 2-phenylacetaldehyde occurs preferentially in berry skin rather than in the juice or pulp. 2-Phenylacetaldehyde has a sweet, honey-like, hawthorn aroma and it has a low odor perception threshold (5 μg/L in model wine) [46,60], suggesting that it may contribute significantly to the aroma of Sabrevois and St. Croix juice.

The varieties Sabrevois and Maréchal Foch showed significant concentrations of short to medium chain FAEE, mainly ethyl butyrate and ethyl 2-butenoate, in both their juice and berry skin. Occurrence of short to medium chain FAEE is unusual in most *V. vinifera* grape varieties but seems to be ubiquitous in *V. labrusca* and its hybrids [49,61,62], which accounts for their presence in Sabrevois that contains a significant proportion of *V. labrusca* in its parentage [6]. The metabolic origins of ethyl esters in *V. labrusca* grape relate to the presence of anthraniloyl-CoA methanol acyltransferase (AMAT), a specific enzyme not found in *V. vinifera* [63] and responsible for the biosynthesis of the “foxy” compound methyl anthranilate as well as other FAEE such as ethyl butyrate [63]. Maréchal Foch, despite the absence of *V. labrusca* in its parentage (50% *V. vinifera*, 25% *V. rupestris* and 25% *V. riparia*), showed significant levels of FAEE in its juice and berry skin. Since short chain FAEE (<C_6_) have not been reported, or found in trace amount in both *V. vinifera* and *V. riparia* [45,58], it could be hypothesized that *V. rupestris* may be responsible for the production of these esters in Maréchal Foch, since this variety contains a significant proportion of *V. rupestris* in its parentage [6,64]. However, to our knowledge, no data is available on the volatile compounds of *V. rupestris*, leaving this hypothesis unanswered. Of interest for grape juice production, most of the short chain FAEE analyzed in the present study, including ethyl propanoate, 2-methylpropanoate, butanoate, 2-methylbutanoate, 2-butenoate and hexanoate, were detected at a concentration above their odor perception thresholds, which range between 0.1 (ethyl 2-methylpropanoate, ethyl 2-methylbutanoate) and 14 μg/L (ethyl 2-butenoate) in water [65,66,67], suggesting that they could be significant contributors to the aroma of Maréchal Foch and Sabrevois juice.

In general, the results for grapes showed significant differences between varieties. However, it should be pointed out that some of these differences could in part relate to the ripening stage of certain varieties, especially Sabrevois and St. Croix. Thus, the occurrence of variations in the volatile composition of grapes during ripening, including interspecific hybrids, is well documented [4,51,53,54]. For example, depending on variety, the level of certain C_6_ alcohols such as hexanol may increase during ripening, whereas that of other C_6_ alcohols such as (*Z*)-3-hexenol, may decrease [4,54]. In the present study, Sabrevois and St. Croix were harvested at lower TSS level (18–19 °Brix) compared to the Frontenac, Maréchal Foch and Marquette varieties, in order to comply with the local commercial practices for these varieties. Hence, at the highest, the TSS level of Sabrevois and St. Croix can reach 21 to 22 °Brix in Québec, but at this level both varieties accumulate unpleasant foxy aroma that translates into wine [6], suggesting that foxy compounds may be formed during the latest ripening stage. We could hypothesize that the level of certain classes of compounds (*i.e.*, C_13_-norisoprenoids) would have been higher in riper Sabrevois and St. Croix grapes. However, the present results strongly agree with previous reports on the volatile composition of grapes from different species, suggesting that the present varietal differences overtook the differences relating to the ripening stages, although such differences should be documented in future work.

### 3.2. Grape to Wine Relationships

From the scientific literature on *V. vinifera* varieties, it is now well known that most wine volatiles are produced during fermentation and are, in this respect, yeast-related; however, the small proportion of volatiles contributed by grapes, either as free or fermentation-released aglycones, is also significant to wine aroma because many of these compounds have very low odor perception threshold, making them the core of wine varietal particularities. In the current study, a similar trend was observed: fermentation products, including FFA, ethyl esters, acetates and alcohols, accounted for more than 98% of total volatile compounds quantified in the wines of the analyzed varieties. In contrast, grape-related FADP accounted for 1% to 2% of total wine volatiles, whereas terpenes and C_13_-norisoprenoids represented less than 0.5% of total volatiles.

**Figure 2:**
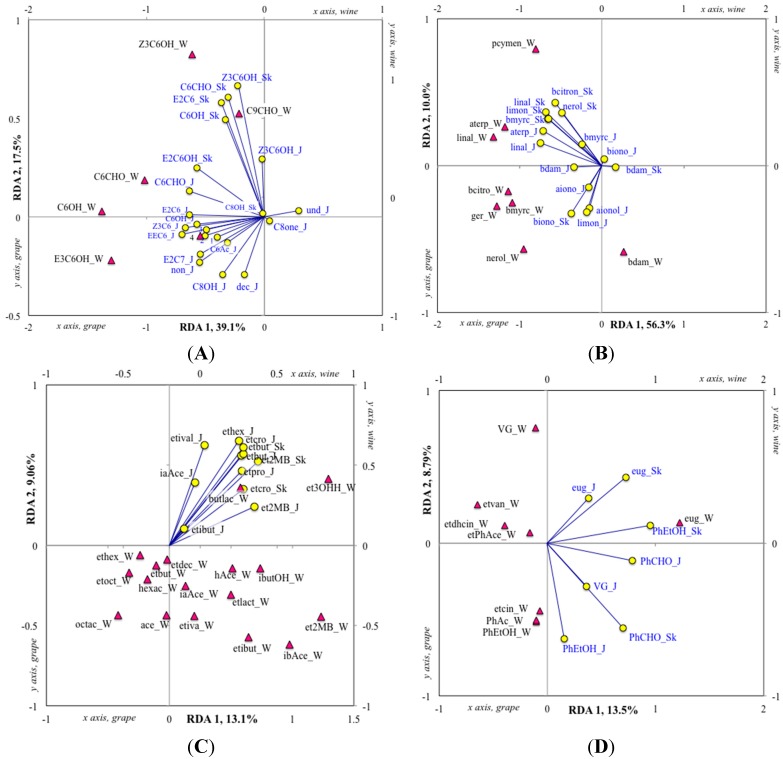
Biplots of four redundancy analyses relating berry volatile compounds (juice and berry skin, independent variables, yellow circles) to wine volatile compounds (dependant variables, red triangles) in the interspecific hybrid grape varieties Frontenac, Marquette, Maréchal Foch, Sabrevois and St. Croix (*n* = 32), for the following groups of compounds: Fatty acid degradation products (**A**), Terpenes and C_13_-norisoprenoids (**B**); Non-aromatic acids, esters, alcohol and acetates (**C**); and Aromatics (**D**). The variables are identified as follow: (**A**) Berry juice (_J) and skin variables (_Sk): hexanal (C6CHO_J; C6CHO_Sk), (*Z*)-3-hexenal (Z3C6_J), (*E*)-2-hexenal (E2C6_J; E2C6_Sk), 2-octanone (C8one_J), (*E*)-2-heptenal (E2C7_J), hexanol (C6OH_J; C6OH_Sk), (*Z*)-3-hexenol (Z3C6OH_J; Z3C6OH_Sk), (*E*,*E*)-2,4-hexadienal (EEC6_J), (*E*)-2-hexenol (juice: 2; E2C6OH_Sk), 1-octen-3-ol (C8OH_J; C8OH_Sk), (*E*,*E*)-2,4-heptadienal (juice: 1), (*E*,*Z*)-2,4-heptadienal (juice: 3), decanal (dec_J), (*E,Z*)-2,6-nonadienal (non_J), 2-undecanone (und_J); Wine variables: hexanal (C6CHO_W), hexanol (C6OH_W), (*E*)-3-hexenol (E3C6OH_W), (*Z*)-3-hexenol (Z3C6OH_W), nonanal (C9CHO_W), (*E*,*Z*)-2,6-nonadienal (wine: 4); (**B**) Berry juice (_J) and skin variables (_Sk): β-myrcene (bmyrc_J; bmyrc_Sk), (*R*)-(+)-limonene (limon_J; limon_Sk), linalool (linal_J; linal_Sk), α-terpineol (aterp_J), β-citronellol (bcitron_Sk), nerol (nerol_Sk), β-damascenone (bdam_J; bdam_Sk), α-ionone (aiono_J), α-ionol (aionol_J), β-ionone (biono_J; biono_Sk); Wine variables: β-myrcene (bmyrc_W), *p*-cymenene (pcymen_W), linalool (linal_W), α-terpineol (aterp_W), β-citronellol (bcitron_W), nerol (nerol_W), geraniol (ger_W), β-damascenone (bdam_W); (**C**) Berry juice (_J) and skin variables (_Sk): ethyl propanoate (etpro_J), ethyl 2-methylpropanoate (etibut_J), ethyl butanoate (etbut_J; etbut_Sk), ethyl 2-methylbutanoate (et2MB_J; et2MB_Sk), ethyl 3-methylbutanoate (etival_J), ethyl (*E*)-2-butenoate (etcro_J; etcro_Sk), ethyl hexanoate (ethex_J), isoamyl acetate (iaAce_J); Wine variables: ethyl 2-methylpropanoate (etibut_W), ethyl butanoate (etbut_W), ethyl-2-methylbutanoate (et2MB_W), ethyl 3-methylbutanoate (etiva_W), ethyl hexanoate (ethex_W), ethyl octanoate (etoct_W), ethyl decanoate (etdec_W), ethyl-3-hydroxyhexanoate (et3OHH_W), hexanoic acid (hexac_W), octanoic acid (octac_W), isobutyl acetate (ibAce_W), isoamyl acetate (iaAce_W), hexyl acetate (hAce_W), ethyl lactate (etlact_W), acetoin (ace_W), butyrolactone (butlac_W), isobutanol (ibutOH_W); (**D**) Berry juice (_J) and skin variables (_Sk): 2-phenylacetaldehyde (PhCHO_J, PhCHO_Sk), 2-phenylethanol (PhEtOH_J; PhEtOH_Sk), eugenol (eug_J, eug_Sk), *p*-vinylguaiacol (VG_J); Wine variables: eugenol (eug_W), *p*-vinylguaiacol (VG_W), ethyl phenylacetate (etPhAce_W), phenethyl acetate (PhAc_W), ethyl dihydrocinnamate (etdhcin_W), ethyl cinnamate (etcin_W), ethyl vanillate (etvan_W), 2-phenylethanol (PhEtOH_W).

Understanding the relationships between grape chemistry and wine sensory perception is one of the ultimate goals of wine science. For wine aroma, the basis of such studies involves relating grape composition, including volatile compounds, to wine volatiles, which constitute an essential part of wine bouquet. In the present work, we studied these relationships by constraining grape variables in redundancy analysis (RDA) models. First, we explored the impact of global grape composition (RDA of volatile compounds groups, Figure 1B; *p* ≤ 0.001, Table 5), and showed a correlation between grape and wine volatiles for certain groups of compounds such as terpenes, whereas limited relationships were noticed for other groups of compounds such as FAEE. Secondly, we analyzed the relationships between particular compounds within specific classes of plant- or fermentation-related compounds (Figure 2, Table 5).

The global RDA showed a strong correlation between the sum of FADP in juice and the sum of FADP in wine, but a weaker correlation was found between the sum of FADP in the skin and the FADP level in the wine (Figure 1B). The RDA model involving the variables comprised in this group (mostly C_6_ compounds; Figure 2A) showed that the variations observed on the canonical axes 1 and 2 were not random (both axis are significant to *p* ≤ 0.001, Table 5) and could explain 56.6% of the variance. However, the RDA model was not significant (*p* ≤ 0.13), suggesting that multiple and variable biochemical and/or chemical reactions may affect the level of FADP in the wine, and that compound-to-compound relationships are not necessarily linear. Hence, it should be pointed out that the compounds comprising the FADP from wines are not exactly the same as those from grapes, as most aldehydes present at high concentrations in grapes are reduced to the corresponding alcohols during the fermentation process, which increases their odor perception threshold, therefore decreasing their sensory impact on the wine [68].

Nevertheless, the RDA of FADP revealed some trends about the presence of FADP compounds with OAVs ≥ 1 in interspecific hybrid wines. For example, the level of hexanal in wine was related to the level of hexanal in the juice. Hexanal reached an OAV ≥ 1 in all wines, with significantly higher concentration in Marquette and Maréchal Foch wines. In contrast, the level of (*Z*)-3-hexenol in wine related to the level of C_6_ in berry skin but not in the juice, suggesting that skin maceration may have an impact on the accumulation of this compound in interspecific hybrid wines. Thus, C_6_ compounds and other FADP are generally assumed to accumulate during the pre-fermentative steps of wine production, especially during grape crushing and maceration with other sources, such as the release of glycosylated precursors during fermentation, having a marginal impact on wine C_6_ level [69]. (*Z*)-3-Hexenol has been reported as an impact odorant in Cabernet Sauvignon and Merlot wines [46], suggesting that it may contribute to the aroma of Marquette wines, where its OAV reached 1. Compared to our data, in berries of similar TA and having slightly higher total soluble solids level, much higher concentrations of (*Z*)-3-hexenol were reported in Maréchal Foch wines from different crop loads (205–1020 μg/L), reaching OAVs ranging from 0.5 to 2.6 [22]. This suggests that, under certain conditions, (*Z*)-3-hexenol may significantly impact on the wines from this variety. The level of (*Z*)-3-hexenol in interspecific hybrids was shown to decrease during berry ripening [4].

Despite their low odor perception threshold, limited attention is usually dedicated to C_9_ aldehydes in wine. In our interspecific hybrid wine samples, nonanal reached significant OAVs (113–144), suggesting that this compound could significantly contribute to hybrid wine aroma. Nonanal has a fatty odor with orange and rose notes on dilution [70]; levels ranging between 4 μg/L and 34 μg/L have been reported in *V. vinifera* wines for this compound [71]. In plants, nonanal mainly arise from the non-enzymatic degradation of 9-hydroperoxides of oleic acid [72]. In our study, the formation of nonanal occurred during winemaking, as nonanal was neither detected in juice nor in berry skin. This suggests that significant levels of oleic acid could be present in interspecific hybrid grapes, possibly in lipids that are not readily accessible in berries. In winemaking, the level of nonanal can be impacted by the yeast and bacteria strains used for the fermentations, and by fermentation temperature [73,74]. Similar to nonanal, the C_9_ aldehyde (*E*,*Z*)-2,6-nonadienal showed OAVs ranging from 30 to 52 in hybrids wines. (*E*,*Z*)-2,6-Nonadienal has a very low odor perception threshold (0.01 μg/L, in water) and is generally described as cucumber-like [34]. Concentration comprised between 0.1 and 12 μg/L have been reported in Italian *V. vinifera* wines [71]. (*E*,*Z*)-2,6-Nonadienal showed higher level in wine compared to juice, suggesting that its formation occurred during winemaking, possibly from glycosylated precursors, or from the enzymatic oxidation of α-linolenic acid, via the 9-hydroperoxide route [72].

When compared to other interspecific hybrid wines produced in this study, Marquette wines showed significantly higher levels of terpenes, including geraniol (19 μg/L, OAV = 1.4) and linalool (36 μg/L, OAV = 0.6), which was consistent with the significantly higher terpene concentrations found in Marquette juice and skin compared to other studied varieties. Thus, the global RDA showed a clear correlation between the level of terpenes in the juice and the skin, and the level of terpenes in the wine (Figure 1B). The RDA analysis of terpenes and C_13_-norisoprenoids (Figure 2B, *p* ≤ 0.001, Table 5) showed that linalool content of wine strongly related to the linalool content of juice and skin. The high concentration of terpene observed in Marquette wine compared to that of juice and skin, suggests that, similar to *V. vinifera* varieties, a significant proportion of grape terpenes is glycosylated and further liberated during winemaking. Because of this characteristic, optimization of terpene liberation using specific winemaking techniques such as yeast selection, exogenous glycosidase addition, and optimized skin contact could contribute to enhance the quality of Marquette wines. Hence, the extent to which terpenes contribute to Marquette wine aroma remains unknown, but it can be hypothesized that both linalool, previously identified as an impact odorant in Frontenac wines [20], and geraniol contribute to the aroma of Marquette wines because their OAVs are higher than 0.5.

Despite the relatively high levels of β-damascenone in the juice and the skin of the interspecific hybrid grapes analyzed (2–6 μg/L in the juice and 12–34 μg/kg berry in skin), all varieties showed similar levels of β-damascenone in the wine. Therefore, little correlation could be established between the β-damascenone content of grapes compared to its content in the wine. The decrease of β-damascenone level during winemaking could be attributable to the addition of sulfite during the winemaking process, since sulfites have been shown to decrease the level of β-damascenone in wine [75]. Nevertheless, the concentration of β-damascenone found in the hybrid wines was higher than its perception threshold of 0.05 μg/L [71], therefore suggesting that this compound contributes significantly to the wine aroma of interspecific hybrid varieties. GC-O/MS analyses conducted in our lab allowed the detection of β-damascenone in Frontenac, Marquette and Maréchal Foch. In contrast, Mansfield (2011) did not report this compound as an impact odorant in Frontenac wines from Minnesota [20]. β-Damascenone has been reported as an impact odorant in the wine of many *V. vinifera* varieties [45,46,76] and some non*-V. vinifera* varieties [17,77], but recent studies suggest that it could rather act as an exhauster for fruity notes in wine, and potentially cover the green notes attributable to herbaceous compounds such as methoxypyrazines [78,79]. Within the varieties analyzed, the highest β-damascenone OAV was found in Frontenac wines (OAV = 80), which are known for their fruitiness including cherry and blackberry notes [21].

In wine, the vast majority of non-aromatic esters, alcohols and fatty acids come from fermentation. Therefore, the level of FAEE in grapes had little impact on the levels of FAEE in interspecific hybrid wines, as demonstrated by the global RDA of grouped compounds, and on the RDA regrouping non-aromatic compounds (Figure 2C, *p* ≤ 0.57, Table 5). Nevertheless, this RDA showed a trend between the presence of FAEE in the juice and berry skin, as observed in Maréchal Foch and Sabrevois, and the occurrence of ethyl 3-hydroxyhexanoate in wine (14 and 19 μg/L, respectively), although its level remained below its perception threshold. On the other side, significantly higher levels of FAEE (4200 μg/L), including ethyl butanoate, ethyl hexanoate, ethyl octanoate and ethyl decanoate, were found in Marquette wines that also showed significantly higher concentration of FFA (3600 μg/L), including hexanoic and octanoic acid, compared to the other varieties. The biosynthesis of FAEE is directly dependent on the availability of FFA, and both depend on fermentation conditions such as yeast strain, nutrient status of the must (e.g., sugar, assimilable nitrogen), and temperature [80]. Although fermentations were conducted using the same protocol for all grape samples, the technological parameters, including sugar/acidity balance, yeast assimilable nitrogen and pH possibly showed optimal values in Marquette juice, which led to higher levels of FAEE and FFA in Marquette wines. The biosynthesis of FFA and FAEE by yeasts had been found to be negatively impacted by the occurrence of grape-derived linoleic acid in the must which is higher in varieties showing high cold-tolerance [81], a trend that the highly cold tolerant variety Marquette [59] does not seem to follow.

Similar to FAEE and other non-aromatic compounds, the global RDA model showed little correlation between the content of aromatic compounds in grape juice and skin, and the occurrence of aromatic compounds in wine. The exception to this is the level of eugenol in wine, which strongly correlated with the level of 2-phenylethanol in berry skin, a trait primarily observed in Sabrevois. Despite relatively low levels of eugenol in their juice (0.8 μg/L) and berries (0.7 μg/kg berry), Sabrevois wines showed particularly high levels of eugenol (23 μg/L, OAV = 4) compared to the other analyzed varieties (4.3–8.4 μg/L), suggesting that significant amount of this compounds could be glycosylated in grapes. Thus, eugenol is known to occur as glycosylated precursor in grapes and may therefore be released during fermentation [82]. In *V. vinifera* wine, eugenol is usually associated with oak maturation, or smoke-taint, an aromatic default occurring when grapes are exposed to significant amount of smoke [83]. Odor-potent levels of eugenol as high as 328 μg/L (OAV = 55) have been reported in wines made from *V. cinerea* but, in a similar range than our results, a concentration of 16 μg/L was found in *V. riparia* wines [15]. Because of its clove-like, smoky aroma [15,83], eugenol could be one of the compounds involved in the so-called “bacon taint” known to occur in certain Sabrevois wines [84]. The aromatic compounds ethyl cinnamate and ethyl dihydrocinnamate reached OAVs comprised between 1 and 4 in the interspecific hybrid wines produced during this study. Both ethyl cinnamate and ethyl dihydrocinnamate have been identified as aroma-active compounds in Pinot Noir wines [85], and their concentration in Pinot Noir wines from Oregon ranged from 1.9 to 6.4 μg/L, and from 0.4 to 1.2 μg/L respectively [86]. The level of ethyl cinnamate ranged from 3.5 (Marquette) and 4.2 μg/L (Frontenac) but no significant differences were observed between the varieties; in contrast, significantly higher level of ethyl dihydrocinnamate was found in Marquette wines (4.8 μg/L). Both ethyl cinnamate and ethyl dihydrocinnamate are thought to occur from acid-catalyzed reactions involving grape-derived precursors, but their biosynthetic origins in wine remains to be determined [87].

## 4. Experimental Section

### 4.1. Grape Samples

Samples from the red *Vitis* spp. varieties Frontenac, Maréchal Foch, Marquette, Sabrevois, and St. Croix were used in this study. Frontenac (Landot 4511 × University of Minnesota *V. riparia* selection no. 89) and Marquette (MN 1094 × Ravat 262) are selections from the University of Minnesota; Maréchal Foch (Millardet et DeGrasset 101-14 × Goldriesling) is a French hybrid selected by Eugène Kuhlmann in 1911; Sabrevois and St. Croix (both varieties are cross of E.S. 283 × E.S. 193) are selections from Elmer Swenson (Minnesota) [88]. Grape samples (50 kg) were collected during the 2012 season, at commercial harvest, in the wine producing areas Ile d’Orléans (46° 51′N, 71° 6′W), Montérégie-Est (45°26′N, 72°53′W), Montérégie-Ouest (45°7′N, 72°48′W), located in the Province of Québec, Canada. In an effort to identify characteristics relating to grape variety rather than growing conditions, grape samples were sourced from various commercial vineyards located in the aforementioned regions. In Québec, most varieties are trained using Vertical Shoot Positioning, except Maréchal Foch that is usually trained using shorter trellis systems such as Guyot, because vines are generally buried, or protected under geotextiles during winter. For each grape variety, six to eight samples were harvested from the different sites. Samples were transported at 4 °C from the vineyards to the research winery. For each sample, one part (15 clusters; 1 kg) was used for berry and juice analysis, a second part (15 clusters; 1 kg) was frozen (−20 °C) for postharvest analyses, and the remaining was use for winemaking. Each grape sample was analyzed and fermented independently from other samples hence providing six to eight wines per variety.

### 4.2. Reagents and Standards

Absolute ethanol was purchased from Commercial Alcohols (Brampton, ON, Canada). l-Tartaric acid and sodium chloride (NaCl) were purchased from Fisher Scientific (Fair Lawn, NJ, USA). Deuterated standards, including ethyl acetate-d_8_, ethyl butanoate-4,4,4-d_3_, benzyl-2,3,4,5,6-d_5_ alcohol, 2-phenyl-d_5_-ethanol, hexanoic-d_11_ acid, ethyl octanoate-d_15_, and hexanol-d_13_ were purchased from C/D/N Isotopes Inc. (Pointe-Claire, QC, Canada). β-Myrcene was purchased from MP Biomedicals (Santa Ana, CA, USA). Ethyl hexanoate and ethyl propanoate were purchased from Nu-Chek-Prep (Elysian, MN, USA). Ethyl vanillate and nonanal and were purchased from Alfa Æsar (Heysam, U.K.). Acetoin, butyrolactone, β-citronellol, *p*-cymenene, β-damascenone, decanal, ethyl butanoate, ethyl cinnamate, ethyl decanoate, ethyl dihydrocinnamate, ethyl-3-hydroxyhexanoate, ethyl 2-methylbutanoate, ethyl 3-methylbutanoate, ethyl 2-methylpropanoate, (−)-ethyl L-lactate, ethyl octanoate, ethyl phenylacetate, ethyl (*E*)-2-butenoate, eugenol, fructose, geraniol, d-(+)-gluconic acid δ-lactone, glucose, (*E*,*E*)-2,4-heptadienal, (*E*,*Z*)-2,4-heptadienal, 1-heptanol, (*E*)-2-heptenal, (*E*,*E*)-2,4-hexadienal, hexanal, hexanoic acid, hexanol, (*Z*)-3-hexenal, (*E*)-2*-*hexenal, (*Z*)-3-hexenol, (*E*)-2-hexenol, (*E*)-3-hexenol, hexyl acetate, α-ionol, α-ionone, β-ionone, isobutanol, isoamyl acetate, isobutyl acetate, linalool, (*R*)-(+)-limonene, nerol, (*E*,*Z*)-2,6-nonadienal, γ-nonalactone, γ-octalactone, octanoic acid, 2-octanone, 1-octen-3-ol, 1-octen-3-one, phenethyl acetate, 2-phenylacetaldehyde, 2-phenylethanol, α-terpineol, 2-undecanone, and *p*-vinylguaiacol were bought from Sigma-Aldrich (St. Louis, MO, USA).

### 4.3. Basic Metrics for Grapes and Juice

For each sample, 200 berries were stemmed from 15 randomly chosen clusters, and weighted to measure berry fresh weight. Grape juice was manually extracted using food-grade polyethylene bags. Total soluble solids (TSS, °Brix), pH and titratable acidity (g/L tartaric acid eq.) were measured according to the methodologies advocated by the American Society for Enology and Viticulture (ASEV), as described by Amerine & Ough [89]. Primary amino nitrogen was determined by UV-Vis spectrophotometry, using an *o*-phthaldialdehyde assay (NOPA) (Unitech Scientific, Hawaiian Gardens, CA, USA); ammonia (NH_3_) and ammonium (NH_4_^+^) were determined by UV-Vis spectrophotometry, using an enzymatic detection assay using α-ketoglutaric acid and reduced nicotinamide adenine dinucleotide phosphate (NADPH) in the presence of l-glutamate dehydrogenase (GDH), to form l-glutamate and oxidized nicotinamide adenine dinucleotide phosphate (NADP+) (Sigma-Aldrich); both values were combined to provide yeast assimilable nitrogen. Analyses were performed in duplicates.

### 4.4. Juice Volatile Compounds Analysis

Volatile compounds from fresh grape juice were extracted by solid-phase microextraction (SPME), and analyzed according to the procedure of Pedneault *et al.* [4], with the following modifications: (1) 5 mL of manually pressed juice was used for SPME samples; (2) a mixture of deuterated standards (50 µL), including ethyl acetate-d_8_, ethyl butanoate-4,4,4-d_3_, benzyl-2,3,4,5,6-d_5_ alcohol, 2-phenyl-d_5_-ethanol, hexanoic-d_11_ acid, ethyl octanoate-d_15_ and hexanol-d_13_, was used as internal standard for calibration, as described below. Internal standards were selected after studying their stability in time and in different matrices, which was achieved by comparing the area of their quant mass between several injections (calibration matrices, juice and wine).

Volatile compounds from juice samples were extracted by solid phase microextraction (SPME), using an autosampler equipped with a thermoregulated agitator and a fiber conditioning station (Gerstel, Linthicum, MD, USA). Extraction was carried out for 25 min, using the conditions described by Pedneault *et al.* [4]. Samples were desorbed for 5 min, in splitless mode, in the inlet of a gas chromatography-mass spectrometry (GC-MS) system (Agilent 6890 Series, Santa Clara, CA) attached to a time-of-flight detector (Pegasus HT TOFMS; Leco, St. Joseph, MI, USA), connected to a computer with the Leco ChromaTOF software (Leco, St. Joseph, MI, USA). The fiber was baked-out at 270 °C for 15 min after each desorption, to avoid carry-over between injections. Compounds were separated on an open tubular DB-Wax column (polyethylene glycol, 60 m × 0.25 mm i.d. × 0.25 μm film thickness; SGE, Austin, TX, USA), in splitless mode. The injector, transfer line, and ion source (70 eV) were maintained at 270, 200, and 200 °C, respectively. The oven temperature was programmed as follows: isothermal at 30 °C for 1 min; increased to 40 °C at a rate of 10 °C/min; increased to 240 °C at a rate of 3.5 °C/min and isothermal for 2 min; and increased to 250 °C at a rate of 20 °C/min and isothermal for 5 min.

Helium was used as the carrier gas under constant flow (1 mL/min). Mass spectra were acquired at a rate of 20 spectra·s^−e^, with a mass range of 35 to 400 *m*/*z* was selected. Targeted analytes were identified by comparing retention time, retention indices and the mass abundance ratios of selected ions with those of authentic standards, and by matching spectral data with the NIST 05 Spectral Library (Table 6).

For quantitation, an 11-point calibration curve was built using authentic standards and deuterated internal standards (Table 6). A matrix based on hybrid grape juice composition was used for calibration [4]. In order to avoid oversaturation of the SPME fiber at the highest calibration levels, maintain constant headspace saturation levels in the vial and keep a constant level of ethanol in the calibration samples, volatile compound standards were separated into two different stock solutions, for which concentration levels in the calibration samples (level 1 to 11) were formulated conversely. For example, in level 1, volatile compound standards from stock solution no 1 where at their maximal concentration, whereas standards from the stock solution no 2 were at their lowest level, and so on until level 11. All standards were analyzed in duplicate. The lowest signal-to-noise ratio used for quantitation was 2.

**Table 6:** Calibration parameters (Retention times (RT), Retention indices (RI), Internal standards, Quantitation masses (*m/z*), Ion ratio masses, Expected ion ratios (*m/z*), Concentration ranges (in μg/L, unless otherwise noted), and Linear regression coefficients (r) for the analysis of volatile compounds in grape juice and berry skin, using GC-MS(TOF)-SPME.

Compound	Absolute R.T. (s)	RI (DB-Wax) ^a^	Internal Standard	Quant Mass (*m/z*)	Ion Ratio Masses (*m/z*)	Expected Ion Ratio	Concentration Range (μg/L)	r
ethyl propanoate	490	951	ethyl butanoate-4,4,4-d_3_	57	57/102	12.3	3–54	0.9826
ethyl 2-methylpropanoate	502	955	ethyl butanoate-4,4,4-d_3_	88	88/116	1.50	4–95	0.9988
ethyl butanoate-4,4,4-d_3_	612			74	74/119	25.6		
ethyl butanoate	617	1028	ethyl butanoate-4,4,4-d_3_	88	88/116	18.3	2–96	0.9997
ethyl 2-methylbutanoate	646	1050	ethyl butanoate-4,4,4-d_3_	102	102/115	7.50	1–53	0.9965
ethyl 3-methylbutanoate	675	1060	ethyl butanoate-4,4,4-d_3_	88	85/88	0.900	2–101	0.9965
hexanal	700	1084	hexanol-d_13_	82	72/82	1.55	141–7053	0.9976
isoamyl acetate	763	1117	ethyl butanoate-4,4,4-d_3_	70	70/87	4.92	0.2–4	0.9958
(*Z*)-3-hexenal	804	1146	hexanol-d_13_	83	69/98	10.3	0.2–8	0.9914
β-myrcene	865	1145	hexanol-d_13_	93	93/136	28.0	2–49	0.9849
ethyl (*E*)-2-butenoate	871	1151	ethyl butanoate-4,4,4-d_3_	99	99/69	0.250	1–50	0.9694
(*R*)-(+)-limonene	945	1201	hexanol-d_13_	136	136/107	0.780	1–55	0.9942
isoamyl alcohol	978	1205	hexanol-d_13_	57	55/70	2.09	0.4–21	0.9974
(*E*)-2-hexenal	978	1220	hexanol-d_13_	98	83/98	3.83	8–397	0.9956
ethyl hexanoate	1014	1220	hexanol-d_13_	88	88/99	2.56	0.2–6	0.9912
2-octanone	1155	1285	hexanol-d_13_	58	71/128	4.49	2–100	0.9990
1-octen-3-one	1186	1313	hexanol-d_13_	97	70/97	5.01	0.5–4	0.9748
(*E*)-2-heptenal	1238	1326^c^	hexanol-d_13_	83	83/112	20.3	0.5–26	0.9993
hexanol-d_13_	1278			64	64/78	3.37		
1-hexanol	1308	1360	hexanol-d_13_	84	69/84	7.20	80–3988	0.9973
(*Z*)-3-hexenol	1374	1391	hexanol-d_13_	67	67/82	3.22	16–834	0.9982
(*E*,*E*)-2,4-hexadienal	1411	1379^c^	hexanol-d_13_	96	95/96	0.311	4–86	0.9936
(*E*)-2-hexen-1-ol	1422	1377	hexanol-d_13_	82	82/100	13.8	10–500	0.9981
ethyl octanoate-d_15_	1467			105	91/105	3.38		
ethyl octanoate	1497	1436	ethyl octanoate-d_15_	101	143/127	0.178	1–57	0.9947
1-octen-3-ol	1520	1465 ^b^	hexanol-d_13_	85	85/99	2.12	2–98	0.9976
1-heptanol	1538	1467	hexanol-d_13_	70	70/83	10.0	10–500	0.9988
(*E*,*Z*)-2,4-heptadienal	1550	1480 ^b^	hexanol-d_13_	81	81/110	6.18	0.5–25	0.9970
(*E*,*E*)-2,4-heptadienal	1621	1482 ^c^	hexanol-d_13_	110	81/95	18.5	3.5–173	0.9930
decanal	1631	1484	hexanol-d_13_	82	82/112	3.30	1–54	0.9891
linalool	1727	1537	hexanol-d_13_	121	121/93	0.183	0.4–21	0.9901
(*E*,*Z*)-2,6-nonadienal	1811	1575	hexanol-d_13_	70	70/94	12.0	0.4–11	0.9918
2-undecanone	1823	1543	hexanol-d_13_	71	71/170	15.1	0.6–30	0.9966
2-phenylacetaldehyde	1912	1625	2-phenyl-d_5_-ethanol	120	91/120	5.06	2–97	0.9964
α-terpineol	2016	1688	benzyl-2,3,4,5,6-d_5_ alcohol	121	121/139	4.48	0.5–5	0.9845
β-citronellol	2165	1762	hexanol-d_13_	95	95/123	2.00	1–4	0.9731
nerol	2229	1770	hexanol-d_13_	123	93/121	0.506	4–20	0.9637
phenetyl acetate	2233	1829	2-phenyl-d_5_-ethanol	104	104/91	4.11	0.1–4	0.9959
hexanoic-d_11_ acid	2251			63	64/93	0.457		
β-damascenone	2254	1836 ^b^	2-phenyl-d_5_-ethanol	121	121/190	4.48	1–50	0.9951
hexanoic acid	2277	1863 ^b^	hexanoic-d_11_ acid	60	60/73	2.18	10–500	0.9916
α-ionone	2334	1830 ^c^	2-phenyl-d_5_-ethanol	192	121/192	9.94	1–25	0.9765
benzyl alcohol-d_5_	2355			96	96/113	0.210		
α-ionol	2391	[1923] ^b^	2-phenyl-d_5_-ethanol	138	138/123	1.74	0.3–25	0.9975
2-phenylethanol-d_5_	2399			96	96/127	4.65		
2-phenylethanol	2402	1925	2-phenyl-d_5_-ethanol	91	91/122	5.43	2–104	0.9886
β-ionone	2468	1947	2-phenyl-d_5_-ethanol	177	177/192	18.5	0.1–3	0.9968
eugenol	2849	2141	2-phenyl-d_5_-ethanol	164	164/149	1.70	0.5–26	0.9803
4-vinylguaiacol	2867	2198	2-phenyl-d_5_-ethanol	150	135/150	1.21	0.05–2	0.9905

^a^ Retention indices were obtained from: Acree & Arn [60], unless otherwise indicated; ^b^ Retention index obtained from El-Sayed, [90]; ^c^ Retention index obtained from Nijssen *et al.* [91].

### 4.5. Berry Skin Volatile Compound Extraction

Randomly selected frozen grapes were weighed (30 g) and thawed at room temperature in a beaker protected from direct light with aluminum foil (approx. 1 h). Once thawed, the grapes were manually peeled to separate the skin from the pulp. Excess juice was removed from the skins by gently patting them with paper towel. The grape skins were weighed and placed in a mortar. Model wine (4 mL, 7 g/L glycerol, 5 g/L tartaric acid, 12% ethanol, pH 3.4 adjusted with 10 N KOH) was added to the skins, along with 20 μL of ascorbic acid (200 g/L, in water) to prevent oxidation [92]. The grape skin mixture was thoroughly crushed and mixed with a pestle for two minutes. A second extraction was carried out by adding another 4 mL of model wine. The mixture was homogenized and allowed to macerate for 5 min, protected from light with aluminum foil. The skin mixture and the first extract were combined (8 mL), transferred to a tube and centrifuged for 5 min, at 4 °C, at a speed of 7232 rcf, using an Eppendorf 5804 R centrifuge (Hamburg, Germany) equipped with a F-34-6-38 rotor. An aliquot (3 mL) of the supernatant was transferred to a 20 mL amber headspace vial containing 3 g of NaCl and 3 mL of deionized water. A solution of internal deuterated standards (50 μL) including ethyl acetate-d_8_, ethyl butanoate-4,4,4-d_3_, benzyl-2,3,4,5,6-d_5_ alcohol, 2-phenyl-d_5_-ethanol, hexanoic-d_11_ acid, ethyl octanoate-d_15_ and hexanol-d_13_, was added. The vial was sealed with a screw cap fitted with a PTFE/silicon septum, vortexed and analyzed immediately by GC-MS(TOF)-SPME. Samples were analyzed and quantified using the same methodology and calibration as described above for grape juice volatile compounds analysis.

### 4.6. Winemaking

Grape samples (50 kg) were destemmed, crushed, treated with potassium metabisulfite (30 mg/L), and put in stainless steel tanks (100 L) equipped with a floating lid. The musts were cold-soaked at 10 °C, for 48 h, allowed to reach 22 °C for yeast inoculation (*Saccharomyces cerevisiae* Lalvin BM 4X4, 250 mg/L, Lallemand, Montréal, QC, Canada) and fermented on skin in a fermentation room maintained at 22 °C, until dryness. Skin extraction was enhanced by punching the marc down twice per day. The alcoholic fermentations were assessed by daily measurement of wines specific gravity (0.990 kg/L) and temperature (°C). The fermentations were completed after seven days. The wines were then pressed, and malolactic fermentation was conducted with *Oenococcus oeni* MBR31 bacteria (inoculated at 200 mg/L, Lallemand, Montréal, QC, Canada) at 23 °C until no more malic acid was detected, using the following method: wine samples were analyzed by thin layer chromatography on silica gel plates (2.5 cm × 7.5 cm × 250 µm film thickness, Silicycle, Québec, QC, USA), using a mobile phase composed of toluene, acetic acid and *n*-butyl acetate (2:1:1), and malic and lactic acids (2 g/L) standards. Results were revealed by spraying the silica plates with a solution of bromophenol blue (1% (*v*/*v*), in ethanol). At the end of malolactic fermentation, wines were racked to stainless steel kegs, sulfited to maintain 30 mg/L free sulfur dioxide, topped with argon gas and stored at 10 °C for 6 months. Wines were then filtered through a 45 µm membrane using a pad filter equipped with a self-prime pump (Buon Vino Super Jet wine filter, Cambridge, ON, Canada), bottled in 750 mL olive-green glass bottles, and stored at 12 °C, in the dark, until analyses, which were carried out six months after bottling.

### 4.7. Wine Composition

Wine pH, titratable acidity and alcohol content were analyzed according to the methodologies advocated by the American Society for Enology and Viticulture (ASEV), as described by Amerine and Ough [89]. Glycerol and volatile acidity were determined by UV-Vis spectrophotometry using the following enzymatic colorimetric assays: Acetic Acid UniFLEX Reagent™ and Glycerol Reagent, respectively (Unitech Scientific). Analyses were performed in duplicates.

### 4.8. Wine Volatile Compounds

Volatile compounds from wine were extracted by solid-phase microextraction (SPME) and analyzed by gas-chromatography-mass spectrometry (GC-MS). Samples were prepared as follows: 3 mL of wine and 3 mL of water were put in amber SPME vials (20 mL) containing NaCl (3 g), and an internal standard mixture (50 µL) constituted of ethyl acetate-d_8_, ethyl butanoate-4,4,4-d_3_, ethyl octanoate-d_15_, benzyl-2,3,4,5,6-d_5_ alcohol, 2-phenyl-d_5_-ethanol, hexanoic-d_11_ acid and *n*-hexyl-d_13_ alcohol. Vials were tightly sealed with a screw cap fitted with a PTFE/silicon septum, homogenized and analyzed immediately, using the GC-MS(TOF)-SPME conditions described earlier for grape volatile compounds.

Volatile compounds from wine were quantitated using an 11-point calibration curve built from authentic standards and deuterated internal standards (Table 7). A wine-like matrix composed of ethanol (10% *v*/*v* in water), glycerol (7 g/L) and tartaric acid (5 g/L), with a pH of 3.4 adjusted with 10 N KOH was used for calibration. In order to avoid oversaturating the SPME fiber and to minimize the impact of alcohol on compounds volatility, calibration samples were formulated conversely using four different stock solutions (authentic standards) to keep ethanol level constant (5% *v*/*v*). All standards were analyzed in duplicates. The lowest signal-to-noise ratio used for quantitation was 2. Odor Activity Values (OAVs) were measured using odor perception thresholds from literature (Table 4), using the following formula: OAV = [analyte]/odor perception threshold, with both terms using the same concentration units (either µg/L or mg/L).

**Table 7:** Calibration parameters [retention times (RT), retention indices (RI), internal standards, quantitation masses (*m/z*), ion ratio masses, expected ion ratios (*m/z*), concentration ranges (μg/L, unless otherwise noted), and linear regression coefficients (r)] for the analysis of volatile compounds in wine using GC-MS(TOF)-SPME.

Compound	Absolute R.T. (s)	RI (DB-Wax) ^a^	Internal Standard	Quant Mass (*m/z*)	Ion Ratio Masses (*m/z*)	Expected Ion Ratio	Concentration Range (μg/L)	r
ethyl acetate-d_8_	392			76	76/96	4.00		
ethyl 2-methylpropanoate	502	951	ethyl acetate-d_8_	71	88/116	2.13	196–24,536	0.9907
isobutyl acetate	597	1015	ethyl acetate-d_8_	86	56/73	1.32	3682–186,471	0.9672
ethyl butanoate-4,4,4-d_3_	612			74	74/119	25.6		
ethyl butanoate	623	1028	ethyl butanoate-4,4,4-d_3_	71	71/116	28.3	60–1537	0.9817
ethyl 2-methylbutanoate	646	1050	ethyl butanoate-4,4,4-d_3_	102	85/102	0.936	2–256	0.9992
ethyl 3-methylbutanoate	675	1060	ethyl butanoate-4,4,4-d_3_	88	57/88	2.98	4–1204	0.9982
hexanal	700	1084	hexanol-d_13_	56	72/82	1.57	3.9–581	0.9983
isobutanol	748	1099	hexanol-d_13_	74	43/74	206	3–268	0.9847
isoamyl acetate	763	1117	ethyl acetate-d_8_	70	70/87	4.01	24.0–722	0.9978
β-myrcene	865	1145	ethyl acetate-d_8_	93	69/93	1.86	0.44–112	0.9901
ethyl hexanoate	1015	1220	hexanol-d_13_	88	88/99	2.56	255–7653	0.9891
hexyl acetate	1109	1270	hexanol-d_13_	61	56/84	3.67	1.07–26.6	0.9993
acetoin	1161	1287	hexanol-d_13_	45	45/88	21.8	493–73,178	0.9965
hexanol-d_13_	1278			64	64/78	3.37		
ethyl lactate	1283	1358	hexanol-d_13_	45	45/75	18.0	579–38,499	0.9956
1-hexanol	1308	1360	hexanol-d_13_	56	56/69	1.40	80–3988	0.9936
(*E*)-3-hexenol	1336	1386	hexanol-d_13_	82	67/82	3.12	0.84–70.2	0.9956
(*Z*)-3-hexenol	1374	1407 ^b^	hexanol-d_13_	67	67/82	1.10	0.34–84.0	0.9938
nonanal	1387	1415 ^b^	hexanol-d_13_	57	98/124	11.1	4.8–98.0	0.9916
ethyl octanoate-d_15_	1467			105	91/105	3.38		
*p*-cymenene	1479	1438 ^d^	hexanol-d_13_	132	117/132	1.13	1.12–75.0	0.9948
ethyl octanoate	1497	1446 ^b^	ethyl octanoate-d_15_	88	101/127	1.82	193–38,122	0.9866
linalool	1727	1537	hexanol-d_13_	136	71/121	7.07	0.50–89.3	0.9913
(*E*,*Z*)-2,6-nonadienal	1811	1575	hexanol-d_13_	41	69/70	1.04	0.22–15.0	0.9964
butyrolactone	1887	[1647] ^c^	hexanol-d_13_	86	56/86	0.996	98.0–540	0.9633
ethyl decanoate	1935	1636	ethyl octanoate-d_15_	88	88/101	2.33	2438–36,570	0.9981
ethyl-3-hydroxyhexanoate	2003	1677	hexanol-d_13_	117	71/117	1.12	1.55–128	0.9992
α-terpineol	2016	1688	benzyl-2,3,4,5,6-d_5_ alcohol	121	121/136	1.39	0.5–24.0	0.9990
β-citronellol	2165	1762	hexanol-d_13_	69	138/156	1.63	0.50–133	0.9958
ethyl-2-phenylacetate	2196	1789 ^d^	benzyl-2,3,4,5,6-d_5_ alcohol	91	91/164	1.48	0.5–129	0.9915
nerol	2229	1782 ^d^	hexanol-d_13_	93	121/136	3.61	1.7–115	0.9955
phenethyl acetate	2233	1808 ^d^	benzyl-2,3,4,5,6-d_5_ alcohol	104	104/91	1.33	0.5–220	0.9929
hexanoic acid-d_11_	2251			46	77/93	6.25		
β-damascenone	2254	1813	2-phenyl-d_5_-ethanol	121	121/190	14.5	0.02–5.00	0.9915
hexanoic acid	2277	1829	hexanoic-d_11_ acid	60	73/87	3.83	100–12 528	0.9920
geraniol	2326	1862 ^b^	2-phenyl-d_5_-ethanol	93	136/154	2.49	0.60–75.2	0.9863
benzyl alcohol-d_5_	2355			113	96/113	0.157		
ethyl dihydrocinnamate	2398	1906	2-phenyl-d_5_-ethanol	104	104/178	2.66	0.10–13	0.9995
2-phenylethanol-d_5_	2399			96	96/127	4.65		
2-phenylethanol	2402	1925	2-phenyl-d_5_-ethanol	91	91/122	4.80	1010–126,332	0.9949
octanoic acid	2678	2083	hexanoic-d_11_ acid	60	73/101	2.44	21.0–20 755	0.9802
ethyl cinnamate	2805	2139	2-phenyl-d_5_-ethanol	131	131/176	4.68	0.02–6.20	0.9954
eugenol	2849	2141	2-phenyl-d_5_-ethanol	164	164/149	1.39	0.4–103	0.9969
4-vinylguaiacol	2867	2198	2-phenyl-d_5_-ethanol	150	135/150	1.21	0.5–66.5	0.9952
ethyl vanillate	3524	2665 ^b^	2-phenyl-d_5_-ethanol	151	151/196	3.46	0.10–37.0	0.9921

^a^ Retention indices were obtained from: Acree & Arn [60], unless otherwise indicated; ^b^ Retention index obtained from El-Sayed, [90]; ^c^ Retention index obtained from Tao & Zhang [93]; ^d^ Retention index obtained from Nijssen *et al.* [91]*.*

### 4.9. Statistical Analysis

Analyses of variance (ANOVA) with a mixed model were performed using the Mixed procedure of the SAS software (Statistical Analysis System Institute, Cary, NC, USA). Means were compared using Tukey’s test at α = 0.05. A redundancy analysis (RDA) was carried out to relate wine volatile compounds (dependent variables) to berry and juice volatile compounds (independent variables), using the R software (The R Foundation for Statistical Computing, Auckland, New Zealand) with the Vegan package. In order to comply with the restriction of the matrix size in R, stating that the number of variables should be equal or lower than the number of samples, variables were grouped by compound types (e.g., FADP, terpenes, FAEE, in juice, skin and wine) and the sums of concentrations were used for the analysis, for a final number of 24 variables for 32 samples. Data were scaled to avoid potential bias related to the wide range of concentrations found for different analytes, some analytes being typically more or less concentrated than others. In order to specify the contribution of each volatile compounds from grape (juice and skin) to the volatile compounds profile of the wine, further redundancy analyses were conducted for four groups of compounds: (1) Fatty acid degradations products; (2) Terpenes and C_13_-norisoprenoids; (3) Non-aromatic esters, alcohols and acetates; (4) Aromatic compounds. Biplots were generated using the BIPLOT.XLA macro for Microsoft Office Excel [94]. The significance of the RDA models was assessed using the anova(rda) function of R, and permutation test (up to 1000 permutations allowed) was used to test the significance of the canonical axes.

## 5. Conclusions

In this work, we reported the volatile composition of the grapes and wines from five interspecific hybrid grape varieties grown in Québec for northern wine production, with an in-depth evaluation of the relationship between grape and wine volatile composition. The chemical composition of the five varieties analyzed showed significant varietal differences even in varieties sharing the same parentage such as Sabrevois and St. Croix. Frontenac, Maréchal Foch and Marquette showed the highest levels of FADP in both grapes and wines whereas Sabrevois and St. Croix showed significantly lower level of FADP in both grape and wines. Maréchal Foch and Sabrevois showed higher levels of FAEE in their juice and berry skin, which related to higher levels of ethyl 3-hydroxyhexanoate in Maréchal Foch and Sabrevois wines. In contrast, the level of β-damascenone in grapes was poorly correlated with that of wine, but its concentration in wine was well over its odor perception threshold, suggesting that this compound contributes to the aroma of interspecific hybrid wines.

Among the five varieties analyzed in this study, Marquette showed valuable characteristics for northern wine production. Thus, Marquette berries had significantly higher levels of terpenes, which correlated with higher levels of terpenes in the wines, including the highly odor potent linalool and geraniol. Similarly, despite the fact that fermentations were carried using a similar protocol for all grape varieties, significantly higher levels of FAEE, FFA and ethyl dihydrocinnamate, were measured in Marquette wines. In addition to its high tolerance to cold temperatures [59], Marquette can reach a suitable sugar/acidity balance for winemaking within a short growing season [4], making it a valuable variety for northern wine production.

The considerable differences between the interspecific hybrid varieties studied suggest various possibilities to enhance hybrid winemaking practices in order to improve northern wine quality. For example, varieties with different FADP levels could be blended to optimized wine aroma and potentially decrease the herbaceousness that may occur in wine. Also, the significant proportion of terpenes in Marquette grapes suggests that specific winemaking techniques such as yeast selection, exogenous glycosidase addition, and optimized skin contact could contribute to enhance the quality of Marquette wines. Similarly, the high level of eugenol in Sabrevois wines suggests that caution should be taken when using oak with this variety, in order to avoid saturating the wine with eugenol. However, further research is needed to provide better understanding of the relationships between wine volatile compounds and the sensory properties of interspecific hybrid wines from northern areas.

## Abbreviations

FW: Fresh weight
GC-MS(TOF)-SPME: Gas Chromatography-Mass Spectrometry (Time-of-flight)-Solid Phase Microextraction
GC-O/MS: Gas Chromatography-Olfactometry/Mass Spectrometry
TA: titratable acidity
TSS: total soluble solids
UV-Vis: UV-visible
RDA: Redundancy analyses
